# Reduced CCL/Be-specific CD4^+^ T cells in CCL3-deficient or peptide-MHCII CAR-T cell–treated mice

**DOI:** 10.1172/jci.insight.195868

**Published:** 2026-06-02

**Authors:** Michael T. Falta, Masoom Raza, Caley J. Nevienski, Tonya M. Brunetti, Rui Fu, Rebecca M. Tucker, Joseph M. Gaballa, Faiz Minhajuddin, Kibrom M. Alula, Alberto Dinarello, Douglas G. Mack, Allison K. Martin, Joseph C. Onyiah, Michael Yarnell, Prashanth Francis, Terry J. Fry, Lisa A. Maier, Andrew P. Fontenot, Charles A. Dinarello, Shaikh M. Atif

**Affiliations:** 1 Department of Medicine and; 2 Department of Immunology and Microbiology, University of Colorado Anschutz, Aurora, Colorado, USA.; 3 New York Genome Center, New York, New York, USA.; 4 Department of Pediatrics, University of Colorado Anschutz Medical Campus, Aurora, Colorado, USA.; 5 Center for Cancer and Blood Disorders, Children’s Hospital Colorado, Aurora, Colorado, USA.; 6 Department of Medicine, National Jewish Health, Denver, Colorado, USA.

**Keywords:** Autoimmunity, Immunology, Adaptive immunity, Autoimmune diseases, T cells

## Abstract

In chronic beryllium disease (CBD), elevated levels of the inflammatory chemokines CCL3 and CCL4 in the lungs coincide with expanded populations of CD4^+^ T cells specific to beryllium-modified (Be-modified) peptides derived from these chemokines. Here, we generated HLA-DP2 transgenic (Tg) CCL3-deficient mice (CCL3^–/–^) that also lack CCL4 to investigate their role in disease development. Be-exposed CCL3^–/–^ mice maintained normal numbers of lung macrophages and dendritic cells (DCs) but exhibited significantly reduced total and HLA-DP2–CCL/Be tetramer-specific CD4^+^ T cells, IFN-γ–producing CD4^+^ T cells, and peribronchovascular aggregates, consistent with attenuated inflammation. CCL3 was predominantly expressed in macrophages and DCs, and bone marrow chimera studies confirm that hematopoietic-derived DCs are the key regulators of CCL/Be-specific CD4^+^ T cell responses. RNA-seq of lung-resident CCL4/Be tetramer^+^ CD4^+^ T cells revealed a transcriptional profile enriched for inflammatory and cholesterol-metabolism pathways, with elevated expression of *Ifng*, *Tnf*, and *Il17a*. Moreover, Be-exposed HLA-DP2 Tg mice lacking TNF-α or treated with peptide-MHCII CAR-T cells targeting CCL4/Be-specific CD4^+^ T cells showed reduced T cell responses and cellular aggregates. These findings demonstrate that CCL3 and CCL4 promote CCL/Be-specific CD4^+^ T cell responses and highlight peptide-MHCII CAR-T cells as a potentially novel strategy for depleting self-peptide/Be-specific CD4^+^ T cells in CBD.

## Abstract 2

Chemokines, CCL3 and CCL4, produced in response to beryllium exposure, are presented as chemokine/Be-neoantigens to CD4+ T cells, inducing pathogenic chemokine-specific CD4^+^ T cell response.

## Introduction

Chronic beryllium disease (CBD) is an occupational disorder characterized by persistent granulomatous inflammation and a pathogenic CD4^+^ T cell response directed against the metal beryllium (Be) (1). More than 1 million individuals are reported to have been exposed to Be^2+^, and of these, a significant fraction (2%–16%) have developed CBD (2). In conjunction with environmental exposure to Be, disease is strongly associated with HLA-DP alleles encoding a glutamic acid at the sixty-ninth position of the β-chain, with HLA-DP2 (HLA-DPB1*02:01) being the most prevalent Glu69-containing allele linked to increased risk of developing CBD (3). The recruitment and expansion of self-peptide/Be-specific CD4^+^ T cells ultimately drive the formation of inflammatory cellular aggregates in the lungs (4–6).

The discovery of Be-modified CD4^+^ T cell epitopes derived from the inflammatory C-C motif chemokines, CCL3, and CCL4 has provided insights into disease pathogenesis. CCL/Be-specific CD4^+^ T cells were enriched in bronchoalveolar lavage (BAL) samples from patients with CBD and are also detected in the lungs of HLA-DP2 transgenic (Tg) mice following exposure to Be. Notably, CCL3 and CCL4 levels were elevated in the BAL of patients with CBD and HLA-DP2 Tg mice (7). Moreover, coadministration of lipopolysaccharide (LPS) with Be further amplified CCL3 and CCL4 secretion, resulting in increased total and CCL/Be-specific CD4^+^ T cells and exacerbated pulmonary inflammation in this mouse model (7, 8). Together, these findings suggest that CCL3 and CCL4 not only contribute to innate immune activation but also serve as a source of neoantigenic targets of the adaptive immune response in CBD (9). To further define their roles in Be-induced immunity and disease development, we generated CCL3^–/–^ HLA-DP2 Tg mice.

Chemokines are key inflammatory mediators involved in many autoimmune and inflammatory diseases (10–13). CCL3 and CCL4, also known as macrophage inflammatory protein-1α (MIP-1α) and MIP-1β, respectively, are distinct yet highly homologous proteins produced by both hematopoietic and nonhematopoietic cells that regulate immune cell migration and function (14–16). CD11c-expressing mononuclear phagocytes, including classical dendritic cells (cDCs), control T cell priming and differentiation through antigen presentation and chemokine secretion (17). In Be-exposed mice, cDCs were shown to be important for promoting effector T cell responses (18). Additionally, Be-treated alveolar macrophages secrete TNF-α, a proinflammatory cytokine known to regulate CCL3 and CCL4 expression in the lungs during asthma and infection (19).

In this study, we used a multipronged approach to investigate the role of CCL3 and CCL4 in the development of granulomatous inflammation in CBD. Using HLA-DP2 Tg WT, CCL3^–/–^ or TNF-α^–/–^ and Zbtb-DTR mice, we found that hematopoietic cells, particularly DCs and macrophages, were the key source of chemokines during Be exposure, and we found that overall innate responses were unaffected by the absence of these chemokines. In contrast, mice deficient in CCL3 (CCL3^–/–^) or TNF-α exhibited markedly reduced total and antigen-specific CD4^+^ T cell responses, fewer cellular aggregates, and decreased inflammatory cytokine production, suggesting a more profound impact on the adaptive immune response. Additionally, transcriptional profiling revealed distinct gene expression signatures in CCL4/Be-specific CD4^+^ T cells (Tet^+^) and CD44^+^Tet^–^ CD4^+^ T cells (Tet^–^) compared with CD44^–^Tet^–^ CD4^+^ T cells (naive) in beryllium oxide (BeO)-exposed HLA-DP2 Tg mice.

Finally, to explore a targeted alternative to corticosteroids and other nonspecific immunosuppressive therapies for CBD, we developed T cells expressing chimeric antigen receptors (CAR-T cells), which have shown promise in treating various forms of cancer and autoimmune disease (20–23). Considering the precision of CAR-T cells in eliminating disease-specific cells, we engineered murine CAR-T cells expressing a pMHCII ectodomain (HLA-DP2–CCL4/Be) to selectively ablate CCL/Be-specific T cells to treat Be-exposed HLA-DP2 Tg mice. This pMHCII CAR strategy offers a potentially novel, antigen-specific approach for depleting pathogenic CD4^+^ T cells, thus presenting a potential alternative to corticosteroids for treating CBD and other granulomatous lung diseases.

## Results

### Macrophage and DC responses in Be-exposed HLA-DP2–CCL3^–/–^ Tg mice.

**Figure 1:**
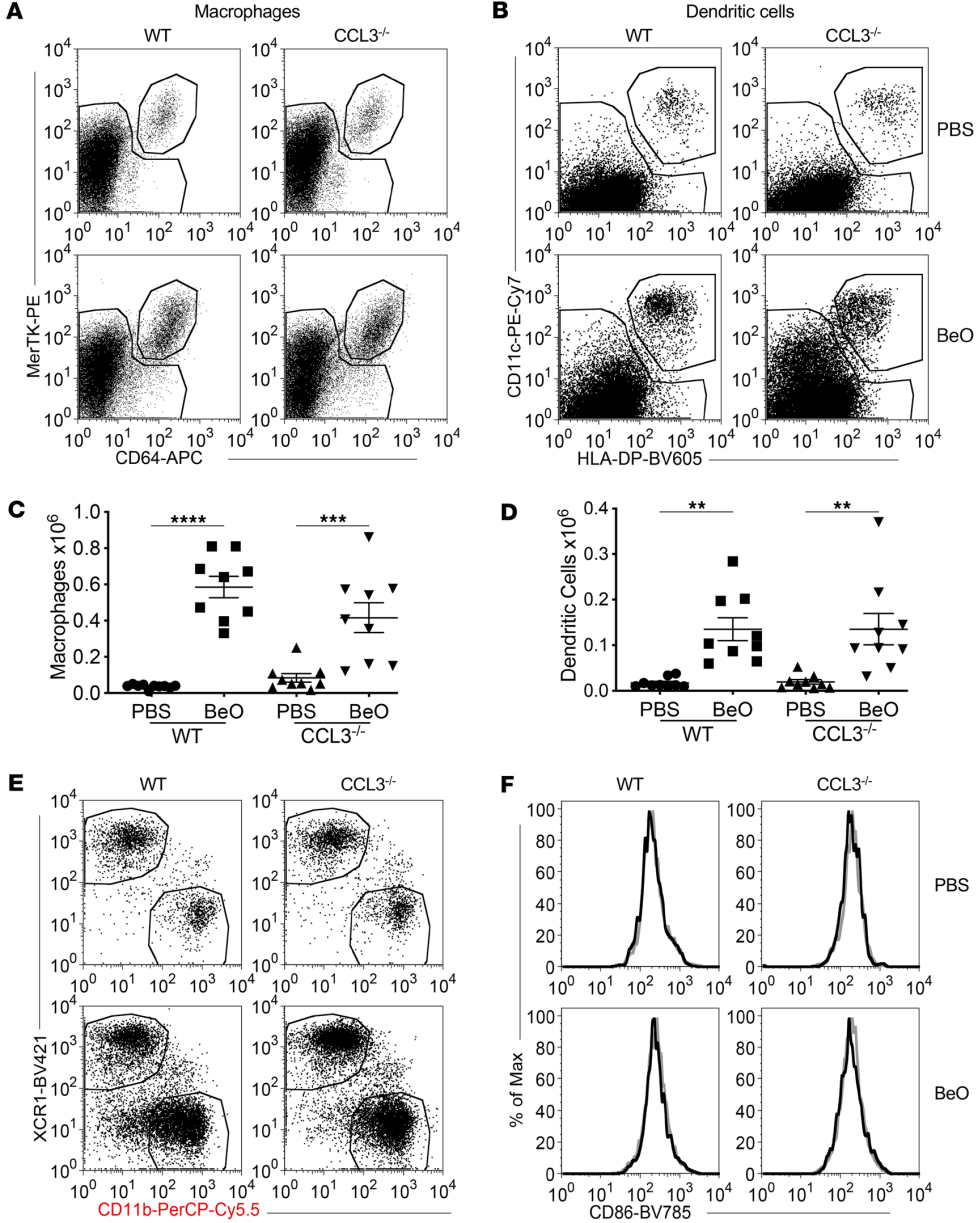
Analysis of innate immune cells in the lungs of HLA-DP2 Tg WT and CCL3-deficient (CCL3^–/–^) mice. HLA-DP2 Tg WT and CCL3^–/–^ mice were intratracheally exposed to PBS or BeO (100 μg) on days 0, 1, and 2 and LPS (10 μg) on day 2. (**A** and **B**) Representative flow cytometric plots show MerTK^+^CD64^+^ macrophages (**A**) and CD11c^+^HLA-DP^+^ dendritic cells (DCs) staining (**B**). (**C** and **D**) Graph plots show the number of tissue-specific macrophages (**C**) and DCs (**D**) in the lungs 24 hours after the last exposure. (**E**) XCR1^+^ (cDC1) and CD11b^+^ (cDC2) DC subsets were examined in the lungs of PBS- (top) and BeO- (bottom) treated WT and CCL3^–/–^ mice. (**F**) Histograms show representative expression of CD86 on cDC1 (gray) and cDC2 cells (black). Each data point represents an individual mouse, and values are shown as (mean ± SEM) combined from 2 independent experiments. 1-way ANOVA determined statistical significance. ***P < 0.001, ****P < 0.0001.

In CBD, the innate immune mediators CCL3 and CCL4 are produced in the lungs in response to Be exposure (7). To investigate their role in Be-induced innate and adaptive immune responses, we generated CCL3^–/–^ HLA-DP2 Tg mice by crossing HLA-DP2 Tg mice with CCL3^–/–^ mice. CCL3^–/–^ mice are hypomorphic for CCL4 (24), and qPCR and ELISA data confirmed the absence of both CCL3 and CCL4 gene and protein expression (Supplemental Figure 1, A and B; supplemental material available online with this article; https://doi.org/10.1172/jci.insight.195868DS1) in HLA-DP2 CCL3^–/–^ Tg mice exposed to BeO/LPS. To assess the impact of CCL3 and CCL4, we first examined pulmonary macrophages and DCs in WT and CCL3^–/–^ mice following short-term exposure to BeO on days 0, 1, and 2 and LPS on day 2. On day 3, leukocytes in the lung tissues were analyzed after excluding the cells in circulation labeled with intravenously injected anti-CD45 mAb. Both WT and CCL3^–/–^ mice showed a similar frequency and number of MerTK^+^CD64^+^ macrophages (Figure 1, A and C) and CD11c^+^MHCII^+^ DCs (Figure 1, B and D) in the lungs. Furthermore, CCL3^–/–^ mice showed no differences compared with treated WT mice in lung cDC1 and cDC2 subsets, and their expression of the costimulatory molecule CD86 (Figure 1, E and F). Thus, these data suggest that the absence of CCL3 and CCL4 does not affect the phenotype of these innate immune cells nor their initial response to pulmonary BeO/LPS exposure.

### Reduced CD4^+^ T cell responses in BeO-exposed HLA-DP2 Tg mice lacking CCL3.

**Figure 2:**
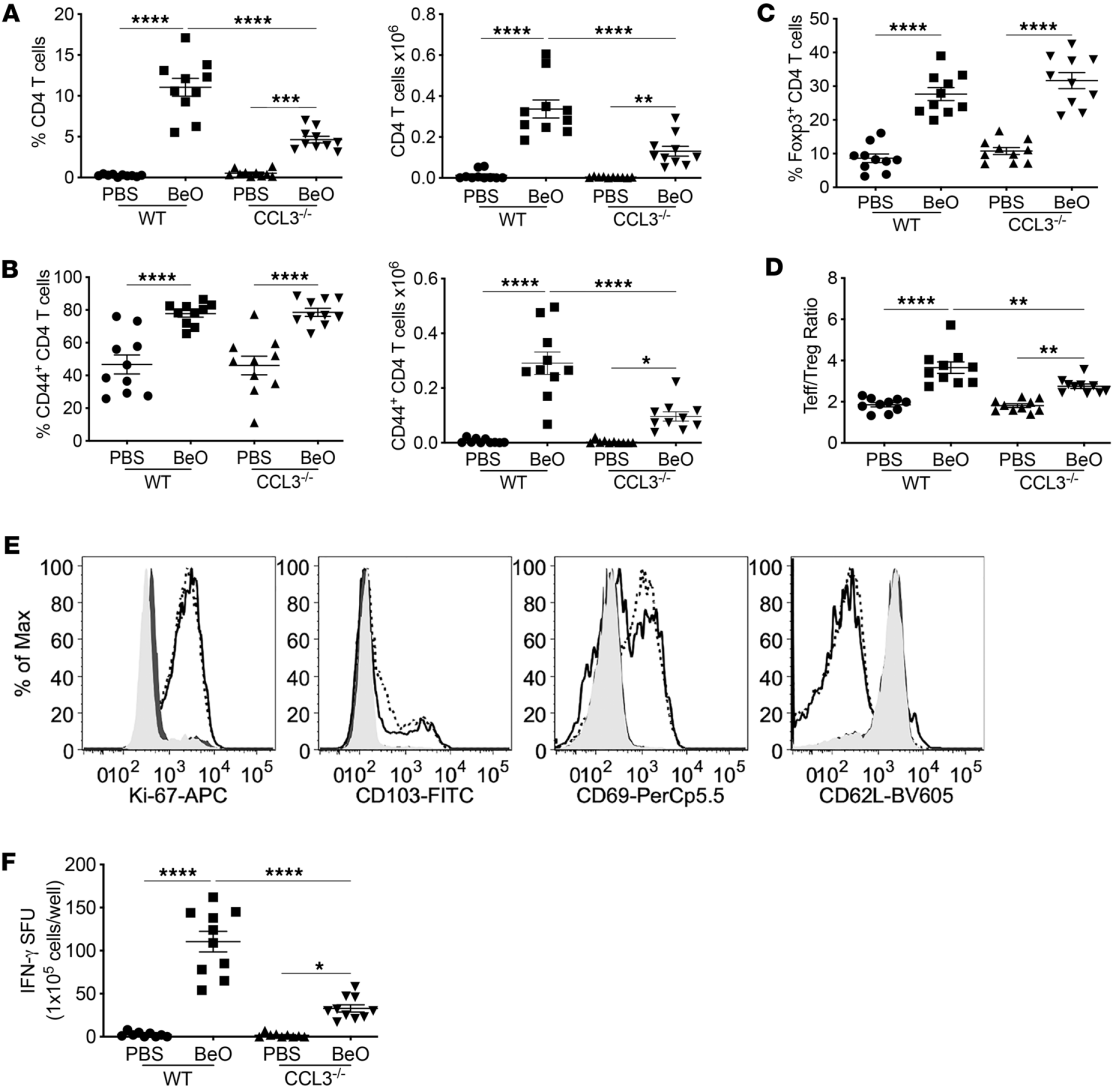
Phenotype of tissue-resident CD4^+^ T cells in BeO-exposed HLA-DP2 Tg CCL3^–/–^ mice. HLA-DP2 Tg WT and CCL3^–/–^ mice were intratracheally exposed to PBS or BeO (100 μg) on days 0, 1, 2, 14, 15, 18, and 19, and LPS (10 μg) was administered on day 14. Mice were sacrificed on day 21. (**A**–**C**) Data plots of isolated lung cells show: (**A**) the frequency (left) and number (right) of tissue-resident CD4^+^ T cells, (**B**) the frequency (left) and number (right) of CD44^+^ activated CD4^+^ T cells, and (**C**) the frequency of FoxP3^+^CD4^+^ T cells. (**D**) Comparison of the Teff/Treg ratio on day 21 of PBS- and BeO/LPS-exposed mice. (**E**) Histograms illustrate the phenotype of CD4^+^ T cell proliferation (Ki-67 staining), tissue-resident (CD103, CD69), and activated (CD62L) cells. Solid histograms show PBS-treated WT (light gray) and CCL3^–/–^ (dark gray) mice; lines show BeO-exposed WT (solid line) and CCL3^–/–^ (dotted line) mice. (**F**) IFN-γ spot-forming units (SFUs) were examined by ELISPOT assay. Each data point represents an individual mouse, and values are shown as (mean ± SEM) combined from 2 independent experiments. 1-way ANOVA determined statistical significance. *P < 0.05, **P < 0.01, ***P < 0.001, ****P < 0.0001.

CD4^+^ T cells play a central role in the pathogenesis of CBD by responding to Be-associated antigens, releasing inflammatory cytokines, and driving lung granuloma formation (25, 26). To assess the absence of CCL3 and CCL4 chemokines on the global CD4^+^ T cell response, HLA-DP2 Tg WT and CCL3^–/–^ mice were sensitized and boosted with BeO (100 μg) and a single dose of LPS (10 μg). On day 21, both the frequency and number of CD4^+^ T cells were reduced by more than 50% in the lungs of CCL3^–/–^ mice compared with WT mice (Figure 2A). Furthermore, although the frequency of CD44^+^ activated CD4^+^ T cells did not differ from WT mice (Figure 2B), the CCL3^–/–^ mice had a significantly reduced number of these T cells (Figure 2B). In BeO/LPS-exposed CCL3^–/–^ mice, the frequency of Tregs in the lungs was higher compared with WT mice, although this difference did not reach statistical significance (Figure 2C). However, consistent with our previous finding that the Teff/Treg ratio correlates with increased lung inflammation due to Be exposure (8), the Teff/Treg ratio was significantly lower in BeO/LPS-exposed CCL3^–/–^ mice compared with WT controls (Figure 2D).

Next, we assessed whether CCL3 deficiency affected CD4^+^ T cell proliferation by staining lung cells for Ki-67, a nuclear protein marker of actively dividing cells (27). Be-exposed WT and CCL3^–/–^ mice showed a similar increase in Ki^+^CD4^+^ T cells (Figure 2E). We also compared the expression of T cell tissue-resident activation and memory markers in WT and CCL3^–/–^ mice. Compared with PBS-treated controls, both WT and CCL3^–/–^ mice showed a similar loss in CD62L expression and similar increases in CD103 and CD69 expression, confirming their tissue-resident phenotype (Figure 2E). Finally, we quantified the number of IFN-γ–secreting CD4^+^ T cells in the lungs of WT and CCL3^–/–^ mice exposed to PBS or BeO/LPS. Following exposure, CCL3^–/–^ mice exhibited approximately a 2.5-fold reduction in IFN-γ–secreting cells compared with WT mice, whereas IFN-γ–secreting cells were absent in PBS-treated WT and CCL3^–/–^ mice (Figure 2F). In contrast, purified CD4^+^ T cells stimulated with the mitogen phytohemagglutinin (PHA) showed no difference between WT and CCL3^–/–^ mice (Supplemental Figure 2), indicating that the reduced IFN-γ response in Be/LPS-exposed CCL3^–/–^ mice is due to the absence of CCL3 and CCL4 rather than a global defect in CD4^+^ T cell function. Collectively, our data suggest that CCL3 and/or CCL4 are required for a robust CD4^+^ T cell response in the lungs of BeO-exposed mice.

### Reduced pathogenic CD4^+^ T cell response in the lungs of BeO-exposed CCL3^–/–^ mice at day 12.

In our standard 21-day BeO/LPS exposure protocol, CCL3^–/–^ mice showed reduced T cell–specific responses compared with WT mice. To investigate the early effect of Be exposure on CD4^+^ T cell recruitment and function, HLA-DP2 Tg WT and CCL3^–/–^ mice were analyzed on day 12 using a modified protocol (Supplemental Figure 3A). CCL3^–/–^ mice exposed to BeO on days 0, 1, and 2 and treated with LPS on day 8 showed no difference in the total number of lung cells compared with WT mice (Supplemental Figure 3B), nor was there any difference observed between PBS-exposed WT and CCL3^–/–^ control mice. However, both the frequency and number of CD4^+^ T cells were reduced in BeO/LPS-exposed CCL3^–/–^ mice compared with WT mice, with no statistically significant differences observed between the treated WT and CCL3^–/–^ mice (Supplemental Figure 3C). Analysis of CD4^+^ T cells showed a similar number of activated CD44^+^ CD4^+^ T cells in the lungs of WT and CCL3^–/–^ mice in response to BeO/LPS (Supplemental Figure 3D).

To assess CD4^+^ T cell functional responses on day 12, ELISPOT assays were performed on purified CD4^+^ T cells from BeO/LPS-exposed mice. Following ex vivo stimulation with BeSO_4_, WT lung CD4^+^ T cells showed a 5-fold increase in IFN-γ–secreting cells and a 4-fold increase in IL-17A–secreting cells compared with CD4^+^ T cells isolated from CCL3^–/–^ mice (Supplemental Figure 3, E and F). CD4^+^ T cells from PBS-exposed WT and CCL3^–/–^ mice did not respond to Be stimulation. Finally, to confirm that reduced T cell responses in CCL3^–/–^ mice were not due to defects in lung T cell homing, we examined expression of the homing receptor CCR7 (C-C chemokine receptor type 7) on tissue-resident CD4^+^ T cells (28). Flow cytometric analysis revealed similar baseline levels of CCR7 expression in PBS-exposed WT and CCL3^–/–^ mice. Upon BeO exposure, both groups exhibited a comparable increase in CCR7 expression (Supplemental Figure 3, G and H), indicating that CCL3-deficiency does not impair T cell homing.

Next, we examined CD4^+^ T cell homing in a non-Be inflammatory setting. WT and CCL3^–/–^ mice were exposed to *Aspergillus nidulans* conidia (1 × 10^8^) via intratracheal administration on day 0, and CD4^+^ T cell frequencies and numbers were determined on day 14. Both WT and CCL3^–/–^ mice exposed to *A*. *nidulans* showed comparable recruitment of CD4^+^ T cells to the lungs (Supplemental Figure 4, A and B). Together, these findings indicate that CCL3 and CCL4 do not affect CD4^+^ T cell homing to the lung but rather play an important role in the development of pathogenic CD4^+^ T cells in CBD.

### Absence of CCL/Be-specific CD4^+^ T cells and cellular aggregates in the lungs of BeO-exposed CCL3^–/–^ mice.

**Figure 3:**
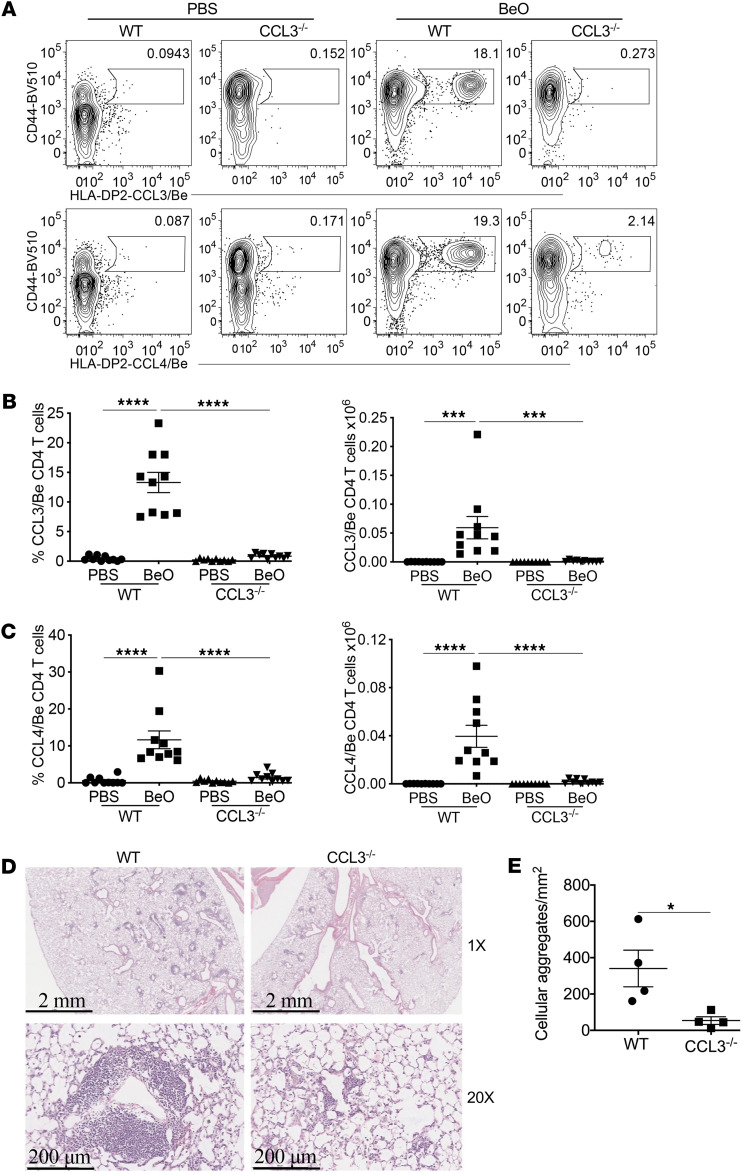
HLA-DP2 tetramer staining of CD4^+^ T cells in BeO/LPS-exposed WT and CCL3^–/–^ mice. HLA-DP2 Tg WT and CCL3^–/–^ mice were intratracheally exposed to PBS or BeO (100 μg) on days 0, 1, 2, 14, 15, 18, and 19 as well as a single dose of LPS (10 μg) on day 14. Lung cells were examined on day 21. (**A**) Contour plots show PE-labeled HLA-DP2–CCL3/Be tetramer (top) and HLA-DP2–CCL4/Be tetramer staining (bottom) of tissue-resident CD4^+^ T cells. (**B** and **C**) Frequency (left) and number (right) of HLA-DP2–CCL3-Be tetramer^+^ (**B**) and HLA-DP2–CCL4/Be tetramer^+^ (**C**) tissue-resident CD4^+^ T cells. Each data point represents an individual mouse; values are shown as (mean ± SEM) from 2 combined independent experiments. One-way ANOVA determined statistical significance. (**D**) H&E staining showing low (1X) and high (20X) magnification of lung tissues. (**E**) Representative quantification of cellular aggregates in WT and CCL3^–/–^ BeO-exposed lungs examined on day 21. A student’s nonparametric 2-tailed *t* test determined statistical significance. *P < 0.05, ***P < 0.001, ****P < 0.0001.

BeO exposure induces robust Be-dependent chemokine peptide-specific CD4^+^ T cell responses in both mice and patients with CBD expressing HLA-DP2 (7, 8). Previously, we also observed a significant enhancement in the chemokine-specific T cell response following exposure to the TLR4 ligand LPS, likely driven by the increased presence of CCL3 and CCL4 as antigens in the lung microenvironment associated with LPS stimulation (7). We hypothesized that mice genetically deficient in CCL3, which also lacks functional CCL4, would fail to develop chemokine-specific T cell responses and have diminished disease pathology. To test this, we used HLA-DP2 tetramers to track HLA-DP2–CCL3/Be and HLA-DP2–CCL4/Be antigen-specific CD4^+^ T cell responses in HLA-DP2 Tg WT and CCL3^–/–^ mice sensitized and boosted with BeO and a single dose of LPS. Prior to assessing tetramer-binding in vivo, tetramer specificity was validated by staining TCR-transduced hybridomas specific for CCL4/Be or CCL3/Be, as well as lung CD4^+^ T cells from BeO-exposed mice. CLIP-loaded HLA-DP2 tetramers were used as a negative control to define background staining (Supplemental Figure 5). Representative HLA-DP2 tetramer staining of experimental mice is shown in Figure 3A. In stark contrast to the high frequency and number of HLA-DP2 tetramer-binding CD4^+^ T cells in BeO-exposed WT mice, there was a pronounced absence of tetramer^+^ cells in the lungs of CCL3^–/–^ mice and the control mice treated with PBS (Figure 3, B and C).

A key feature of the HLA-DP2 Tg mouse model of CBD is the formation of extensive peribronchovascular cellular infiltrates in the lungs after Be^2+^ exposure, and the number and size of these aggregates correlate with the dose of Be^2+^ (8, 29). To determine how the lack of CCL3/4 affects the formation of cellular aggregates, we performed H&E staining on lung tissue from BeO/LPS-exposed mice. Analysis of tissue sections showed greatly reduced peribronchiolar and perivascular mononuclear infiltrates in the lungs of CCL3^–/–^ BeO-treated mice compared with the WT mice (Figure 3D). The cellular aggregates were reduced by more than 8-fold in the CCL3^–/–^ mice as examined in Qupath software (Figure 3E). These data strongly implicate that the adaptive immune response directed against Be-modified CCL3 and CCL4 antigens is critical for the development of lung inflammation in our animal model of CBD.

### Pulmonary DCs control the chemokine/Be-specific CD4^+^ T cell response in the lungs.

**Figure 4:**
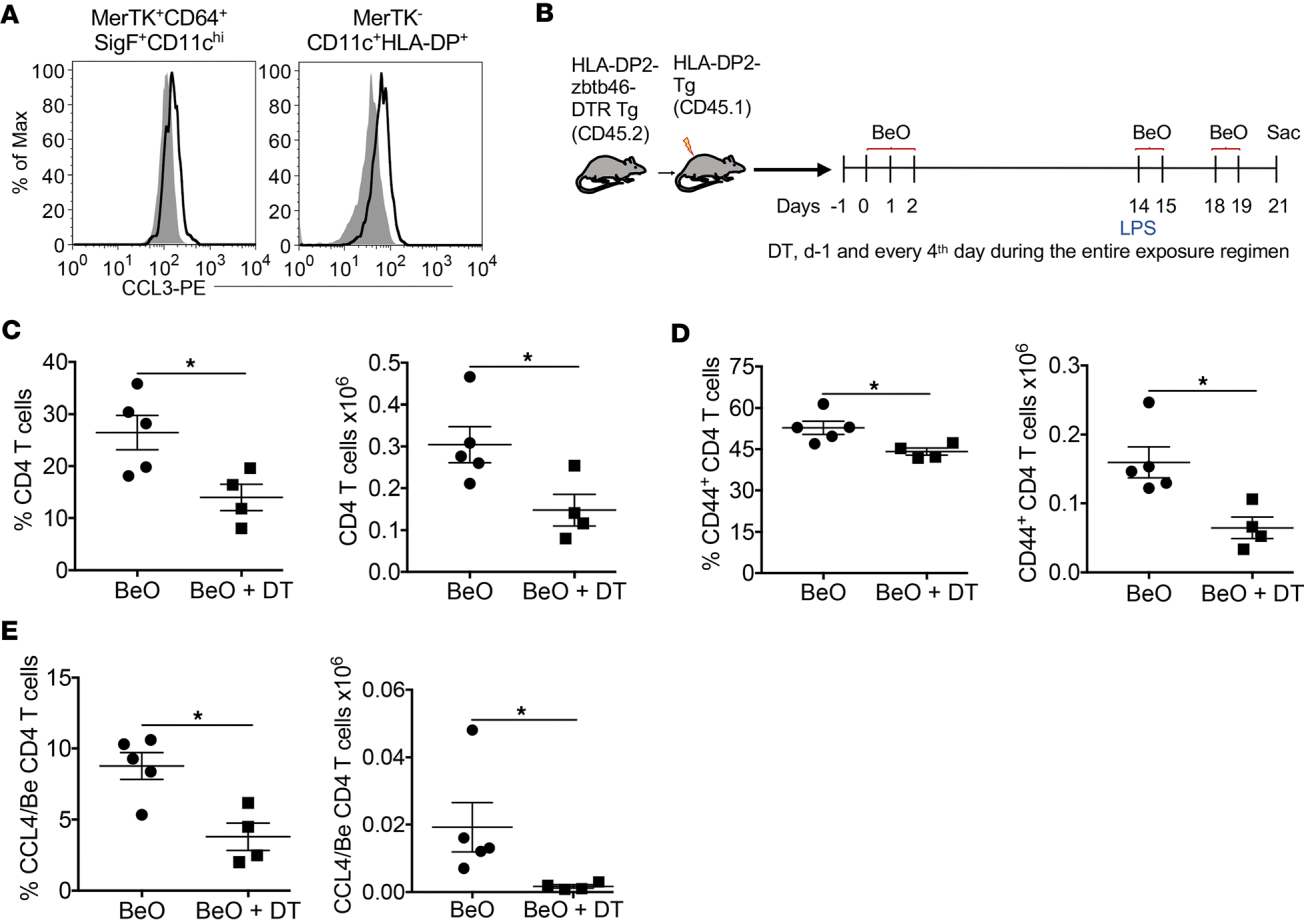
Classical dendritic cells are required for optimal CCL/Be-specific CD4^+^ T cell responses in BeO-exposed HLA-DP2 Tg mice. (**A**) Representative histograms show intracellular staining of CCL3 in lung myeloid cells from HLA-DP2 Tg mice after PBS (solid-gray) or BeO/LPS (black line) standard exposure regimen. On day 21, mice were treated with intratracheal brefeldin A and LPS (10 μg) for 4 hours before sacrifice. (**B**) Diagram illustrates the strategy for assessing the role of conventional dendritic cells in the CCL/Be-specific CD4^+^ T cell response. Eight weeks after reconstitution, 2 groups (*n* = 4–5/group) of lethally irradiated HLA-DP2 Tg CD45.1 mice reconstituted with HLA-DP2–Zbtb46-DTR Tg bone marrow cells were exposed to BeO/LPS. The control group received PBS, and the test group was treated with diphtheria toxin (DT, 5 ng/g body weight) every fourth day, beginning at day –1. (**C**–**E**) Plots show the frequency (left) and number (right) of tissue-resident CD4^+^ T cells (**C**), CD44^+^ activated CD4^+^ T cells (**D**), and HLA-DP2–CCL4/Be tetramer binding CD4^+^ T cells in the lungs of mice exposed to BeO/LPS (**E**) and examined on day 21. Data are representative of 2 independent experiments. A nonparametric Student’s *t* test was used to determine statistical significance. *P < 0.05.

As noted, pulmonary exposure to Be triggers the release of CCL3 and CCL4, and these proinflammatory chemokines control the adaptive immune response in CBD by providing Be-modified neoantigens (7). To identify the cellular source of CCL3, BeO/LPS-exposed HLA-DP2 Tg WT mice were treated with intratracheal Brefeldin A with either PBS or LPS (10 μg) 4 hours before sacrifice. Intracellular staining of isolated lung cells revealed elevated CCL3 levels in both MerTK^+^CD64^+^SigF^+^CD11c^hi^ alveolar macrophages (Figure 4A) and MerTK^–^CD11c^+^HLA-DP^+^ DCs (Figure 4A) compared with other hematopoietic and nonhematopoietic cells (data not shown). Since DCs exhibited higher CCL3 expression and are the most potent antigen-presenting cells (APCs), we investigated whether conventional DCs control CCL3/Be-specific CD4^+^ T cell responses. Bone marrow chimeric mice were generated by transferring bone marrow cells from HLA-DP2–Zbtb46-DTR CD45.2 Tg mice to HLA-DP2 Tg B6 CD45.1 congenic mice (Figure 4B). Zbtb46 is a zinc finger transcription factor specifically expressed in classical DCs among immune lineages (30, 31). After confirming chimerism 8 weeks after transfer, chimeric mice were exposed to BeO/LPS by the standard protocol and treated either with or without diphtheria toxin (DT, 5 ng/g body weight) every fourth day during the entire exposure regimen, beginning at day –1. At sacrifice, mice treated with DT showed a significant decrease in the frequency (Figure 4C) and number (Figure 4C) of lung CD4^+^ T cells. Furthermore, the loss of DCs significantly reduced the accumulation of CD44^+^ effector T cells in the lungs of BeO-exposed mice (Figure 4D). Finally, BeO-exposed mice treated with DT showed a > 2.5-fold decrease in the frequency (Figure 4E) and total number (Figure 4E) of CCL4/Be-specific CD4^+^ T cells in the lungs as measured by HLA-DP2–CCL4/Be tetramer staining. A similar response for HLA-DP2–CCL3/Be-specific CD4^+^ T cells would be expected in these mice, given that CCL3- and CCL4-specific CD4^+^ T cells were comparable in Be-exposed WT mice (Figure 3). Overall, the data suggest an important role for DCs in establishing and maintaining CCL/Be-specific CD4^+^ T cell responses.

### Hematopoietic compartment reconstituted with WT bone marrow restores chemokine/Be-specific CD4^+^ T cell responses in CCL3^–/–^ BeO-exposed mice.

**Figure 5:**
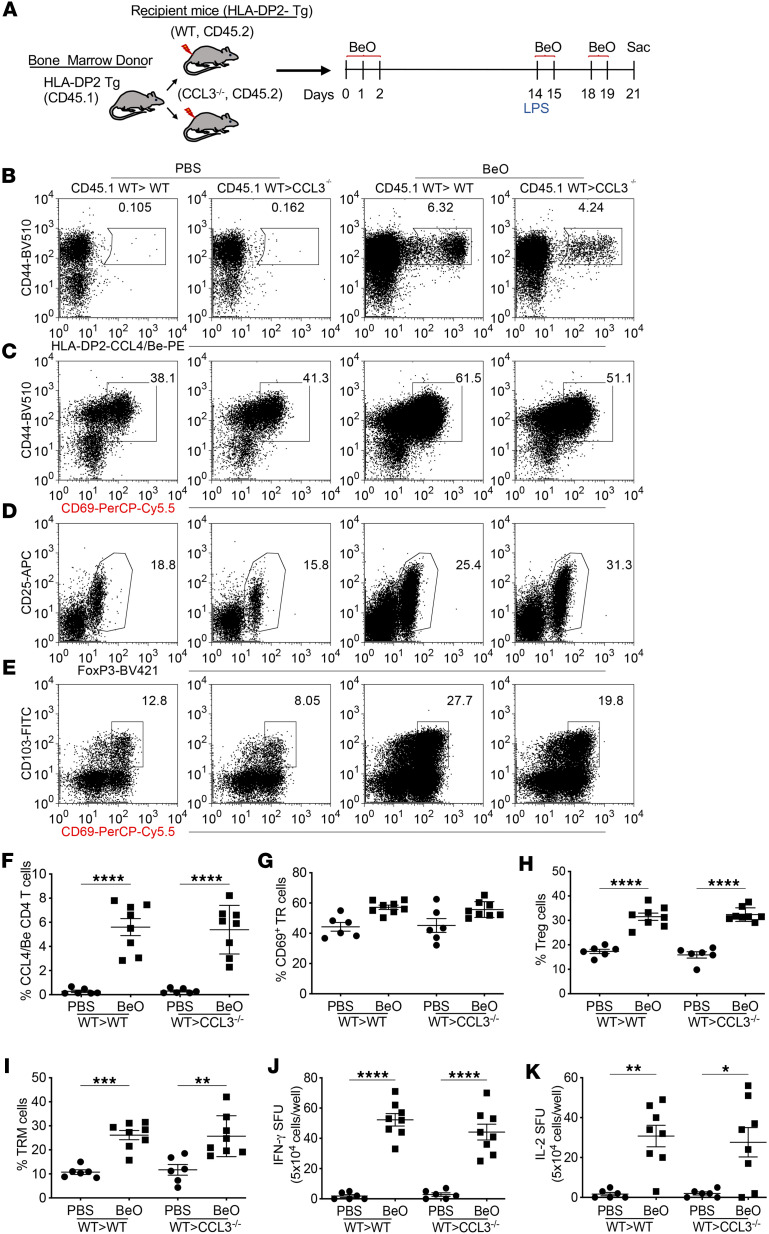
Hematopoietic cells control the CCL/Be-specific CD4^+^ T cell response in the lungs of BeO-exposed mice. (**A**) Diagram shows the generation of bone marrow chimera mice. Lethally irradiated HLA-DP2 Tg WT or CCL3^–/–^ mice were reconstituted with bone marrow cells from congenic (CD45.1) HLA-DP2 Tg mice. (**B**–**E**) Representative flow cytometric plots show the frequency of CCL4/Be-specific (**B**), CD44^+^CD69^+^ tissue-resident (TR) (**C**), CD25^+^FoxP3^+^ Treg (**D**), and CD103^+^CD69^+^ tissue-resident memory (TRM) (**E**) CD4^+^ T cells in the lungs of PBS- and BeO-exposed mice. The recipient mouse strain and treatment received for each column of plots are indicated at the top of **B**. (**F**–**I**) Summary plots of the cell parameters indicated in **B**–**E**. (**J** and **K**) Data plots show IFN-γ (**J**) and IL-2 (**K**) ELISPOT responses as spot-forming units (SFUs) per 100,000 purified CD4^+^ T cells stimulated overnight with BeSO_4_ (100 μM). Each data point represents an individual mouse; values are shown as (mean ± SEM) combined from 2 independent experiments. One-way ANOVA determined statistical significance. *P < 0.05, **P < 0.01, ****P < 0.0001.

Since macrophages and DCs originate from a myeloid lineage, we further examined the role of the hematopoietic compartment in driving chemokine/Be-specific CD4^+^ T cell responses in the lungs of BeO-exposed mice. Lethally irradiated HLA-DP2 Tg WT or CCL3^–/–^ mice (both CD45.2) were reconstituted with bone marrow cells obtained from HLA-DP2 Tg CD45.1 congenic mice. After confirming chimerism, the recipient mice were treated with the standard BeO/LPS regimen (Figure 5A) and analyzed on day 21, after excluding cells in circulation labeled with an intravenously injected anti-CD45 mAb. Both WT and CCL3^–/–^ reconstituted mice showed no difference in the accumulation of HLA-DP2–CCL4/Be-binding CD4^+^ T cells in the lungs of Be-exposed mice (Figure 5, B and F). Additionally, no differences in frequencies were observed in IV-CD45^–^CD4^+^ gated tissue-resident CD44^+^CD69^+^ T cells (Figure 5, C and G), CD25^+^FoxP3^+^ Tregs (Figure 5, D and H), and CD103^+^CD69^+^ tissue-resident memory T cells (Figure 5, E and I). Next, ex vivo IFN-γ and IL-2 ELISPOT assays measuring lung T cell responses to BeSO_4_ stimulation showed no differences between WT and CCL3^–/–^ mice in the number of IFN-γ and IL-2–secreting cells (Figure 5, J and K). Together, these data suggest that cells of hematopoietic origin are the primary cellular sources of CCL3 and CCL4.

### Bulk RNA-seq reveals heterogeneity in antigen-specific tissue-resident CD4^+^ T cells in the lungs of BeO-exposed HLA-DP2 Tg mice.

**Figure 6:**
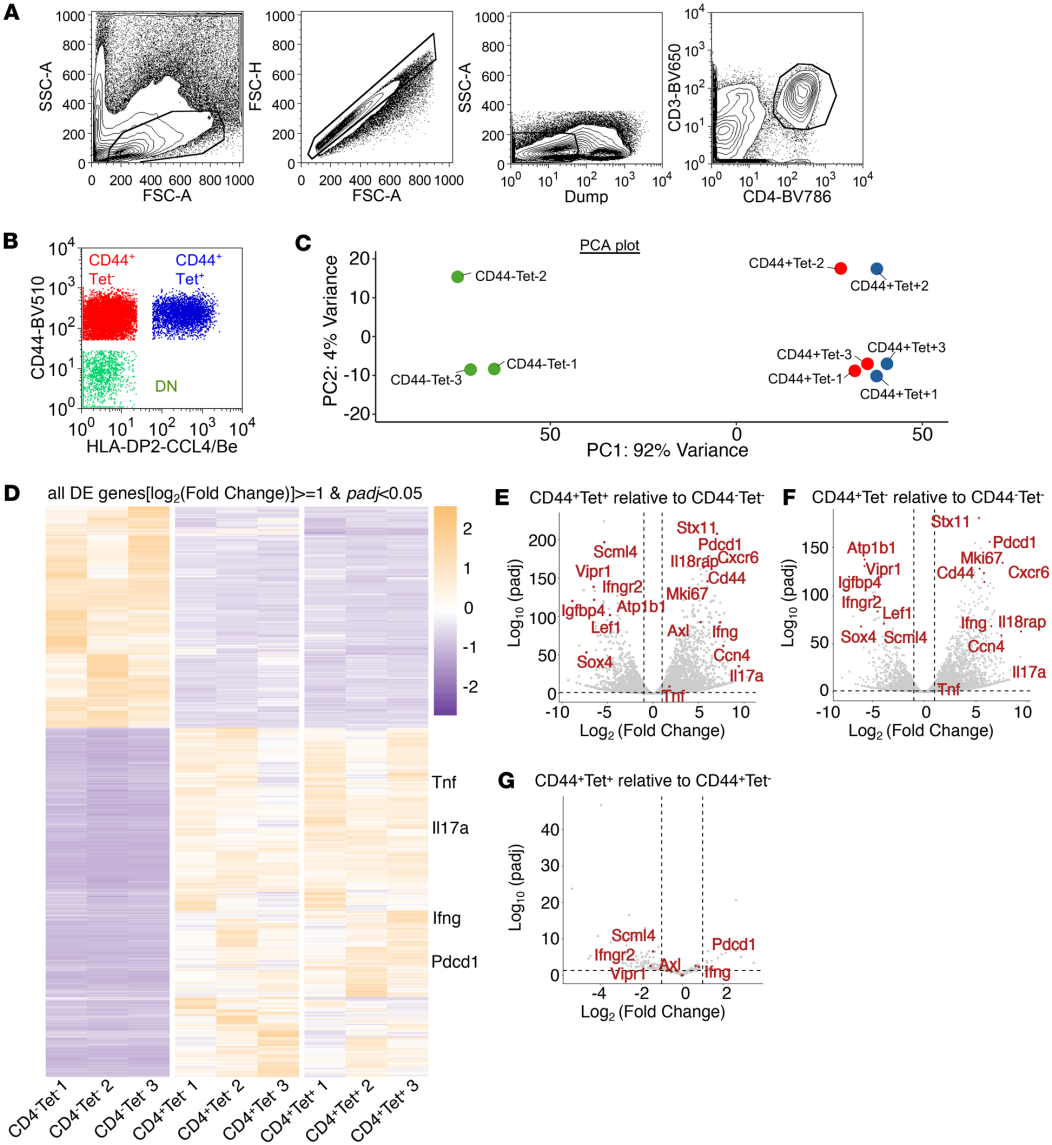
Transcriptional landscape of CD4^+^ T cells in the lungs of BeO-exposed HLA-DP2 Tg mice. HLA-DP2 Tg mice were intratracheally exposed to BeO (100 μg) on days 0, 1, 2, 14, 15, 18, and 19 and a single dose of LPS (10 μg) on day 14. On day 21, 2 minutes before sacrifice, mice were i.v. injected with anti-mouse CD45 mAb (5 μg/mouse) to distinguish circulating T cells from lung-resident T cells. (**A**) Gating strategy to identify lung CD4^+^ T cells after excluding myeloid and B cells in BeO-exposed mice. (**B**) CD4^+^ T cell populations, CD44^+^Tet^+^ cells (Tet^+^, blue), CD44^+^Tet^–^ cells (Tet^–^, red), and CD44^–^Tet^–^ cells (DN, green) flow-sorted from the lungs of BeO-exposed mice. (**C**) Principal component analysis (PCA) plot shows the separation between tissue-resident Tet+ cells (blue), Tet^–^ cells (red), and DN cells (green). (**D**) Heatmap of upregulated (gold) and downregulated (purple) differentially expressed genes between Tet^+^ cells, Tet^–^ cells, and DN cells. Each column in the heat map represents a sample collected from sorted CD4^+^ T cell types isolated from 5 BeO/LPS-exposed mice. (**E**–**G**) Volcano plots showing significantly upregulated and downregulated genes in (**E**) CD44^+^Tet^+^ cells compared with CD44^–^Tet^–^ DN cells, (**F**) CD44^+^Tet^–^ cells compared with CD44^–^Tet^–^ DN cells, and (**G**) CD44^+^Tet^+^ cells compared with CD44^+^Tet^–^ cells.

Similar to CBD (9), CD4^+^ T cells accumulate in the alveolar spaces of HLA-DP2 Tg mice following Be exposure (Figure 6A). Many of these T cells recognize neoantigens composed of Be and peptides derived from CCL3 and CCL4 (7), and include both Th1 and Treg CD4^+^ T cell subsets (8). To explore the transcriptional landscape of CD4^+^ T cells, including CCL/Be-specific CD4^+^ T cells, we performed bulk RNA-seq of lung-resident CD4^+^ T cells from BeO-exposed HLA-DP2 Tg mice to identify unique gene signatures involved in disease pathogenesis that could be therapeutically targeted. We focused on 3 subsets of CD4^+^ T cells: CD44^+^ HLA-DP2–CCL4/Be tetramer^+^ (Tet^+^, blue), CD44^+^ HLA-DP2–CCL4/Be tetramer^–^ (Tet^–^, red), and CD44^–^HLA-DP2–CCL4/Be tetramer^–^ (DN, green) cells (Figure 6B). Principal component analysis (PCA) of differentially expressed genes separated the 2 CD44^+^ subsets from the DN population (Figure 6C). However, the Tet^+^ and Tet^–^ T cell subsets clustered together, suggesting a high degree of overlap in their gene expression profiles despite sorting on a discrete antigen-specific T cell population (Figure 6C). Next, we generated heat maps of the differentially expressed genes in the Tet^+^, Tet^–^, and DN populations. Again, considerable overlap in gene expression was observed between Tet^+^ and Tet^–^ T cells (Figure 6D). The heatmap revealed increased expression of several hundred genes in Tet^+^ and Tet^–^ CD4^+^ T cells compared with the DN populations, including genes associated with activation (*Pdcd1*, *Cd44*, *Il18rap*, *Cxcr6*, *Sell*, *Axl*, *Ccn4*), proliferation (*Mki67*), and effector function (*Ifng*, *Il17*, *Il18*, *Tnf*) (Figure 6, E and F).

To examine whether transcriptional changes were reflected at the protein level, we assessed the expression of selected molecules encoded by genes significantly upregulated in Tet^+^ and Tet^–^ CD4^+^ T cells by flow cytometry. HLA-DP2 Tg mice were exposed to the standard BeO/LPS regimen for 21 days, and lung-resident CD4^+^ T cells were analyzed following HLA-DP2–CCL4/Be tetramer staining. Surface and intracellular staining was performed to measure the expression of PD-1 (*Pdcd1*), CD44 (*Cd44*), CD62L (*Sell*), Ki-67 (*Mki67*), IFN-γ (*Ifng*), TNF-α (*Tnfa*), and IL-17A (*Il17a*). Flow cytometry plots (Supplemental Figure 6A) and mean fluorescence intensity (MFI) values (Supplemental Figure 6B) demonstrated significant differences in expression of these molecules in Tet^+^ and Tet^–^ CD4^+^ T cells compared with DN CD4^+^ T cells.

Additionally, we directly compared the transcriptional profile of Tet^+^ to Tet^–^ CD4^+^ T cells (Figure 6G). In contrast to comparisons with the DN population, few genes showed a greater than 2-fold difference between Tet^+^ and Tet^–^ CD4^+^ T cells. Among these, the *Ifng* gene was upregulated in Tet^+^ relative to Tet^–^ CD4^+^ T cells, and this increase was also noticed at the protein level (Supplemental Figure 6).

In summary, RNA-seq revealed a high level of relatedness in the gene expression profiles of Tet^+^ and Tet^–^ T cells due to their activation status (CD44^+^), consisting of a heterogeneous population of CD4^+^ T cells (Th1, Th17, and Tregs) involved in the pathogenesis of CBD. However, their gene expression profiles were significantly different relative to lung CD4^+^ T cells with a naive phenotype.

### Pathway enrichment analysis reveals increased inflammatory gene expression in CCL4/Be-specific T cells (Tet^+^) compared with naive (DN) T cells.

**Figure 7:**
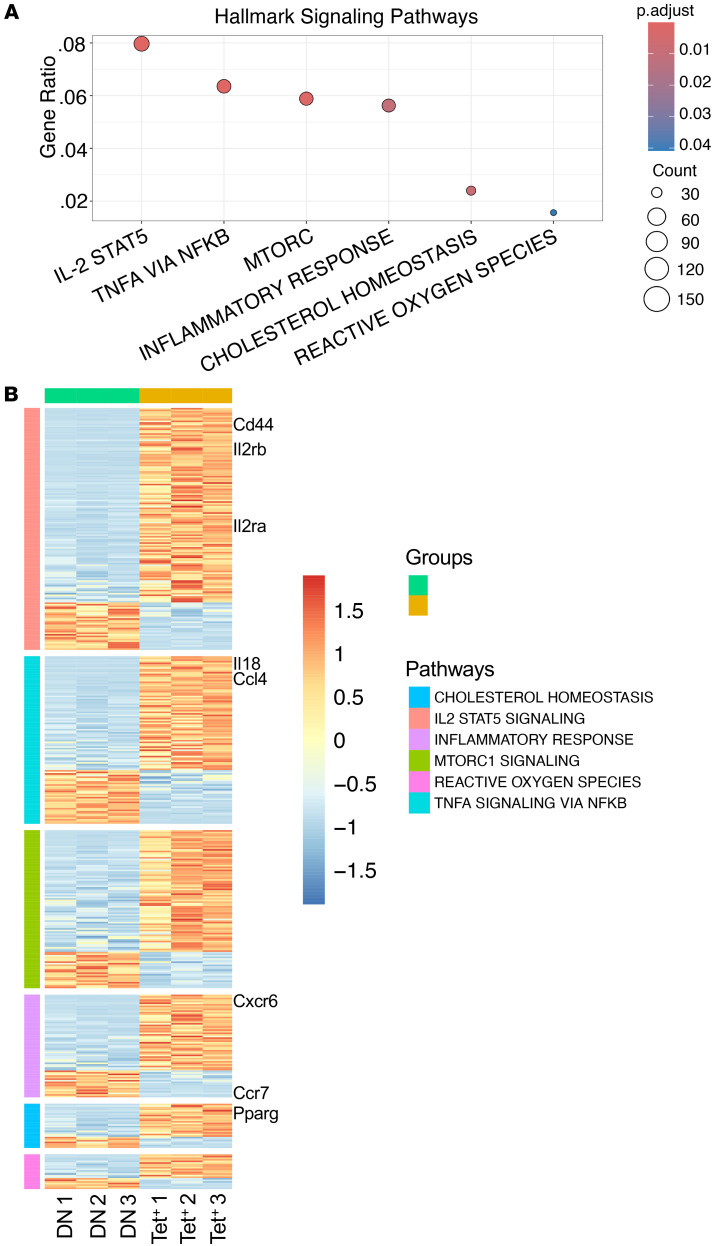
Pathway enrichment analysis of differentially expressed genes in BeO-exposed lung-resident antigen-specific and naive CD4^+^ T cells. HLA-DP2 Tg mice were intratracheally sensitized and boosted with BeO (100 μg) and LPS (10 μg) as indicated in the Figure 6 legend. (**A**) Dot plot showing significantly enriched pathways in antigen-specific CD44^+^Tet^+^ (Tet^+^) CD4^+^ T cells compared with naive (DN) CD4^+^ T cells. (**B**) Heatmap showing genes upregulated and downregulated in the indicated immune and metabolic pathways.

We performed over-representation analysis on differentially expressed genes that were statistically significant (*P*_adj_ < 0.05) and exhibited at least a 2-fold upregulation in Tet^+^ T cells compared with DN T cells. This analysis showed significant enrichment of pathways involved in CD4^+^ T cell development, differentiation, and function, including IL-2/STAT5, TNF-α, and mTORC1 signaling (Figure 7A). The mTORC1 pathway regulates T cell activation, proliferation, and differentiation (e.g., Th1, Th2, and Th17) by integrating signals from antigens, costimulatory molecules, and cytokines, ultimately shaping immune responses and effector functions. Thus, high mTORC1 activity promotes clonal expansion and effector differentiation, while low activity favors Treg development (32). Pathways involved in the inflammatory response and cholesterol homeostasis were also significantly upregulated in Tet^+^ cells compared with DN T cells (Figure 7B). Cholesterol homeostasis maintains normal T cell function, and disruptions in this pathway can impair T cell activation, differentiation, and cytokine production. Together, these data suggest that, in BeO-exposed HLA-DP2 Tg mice, multiple pathways regulate antigen-specific CD4^+^ T cell responses.

### Be-specific CD4^+^ T cell responses are reduced in BeO-exposed HLA-DP2 Tg TNF-α deficient mice.

**Figure 8:**
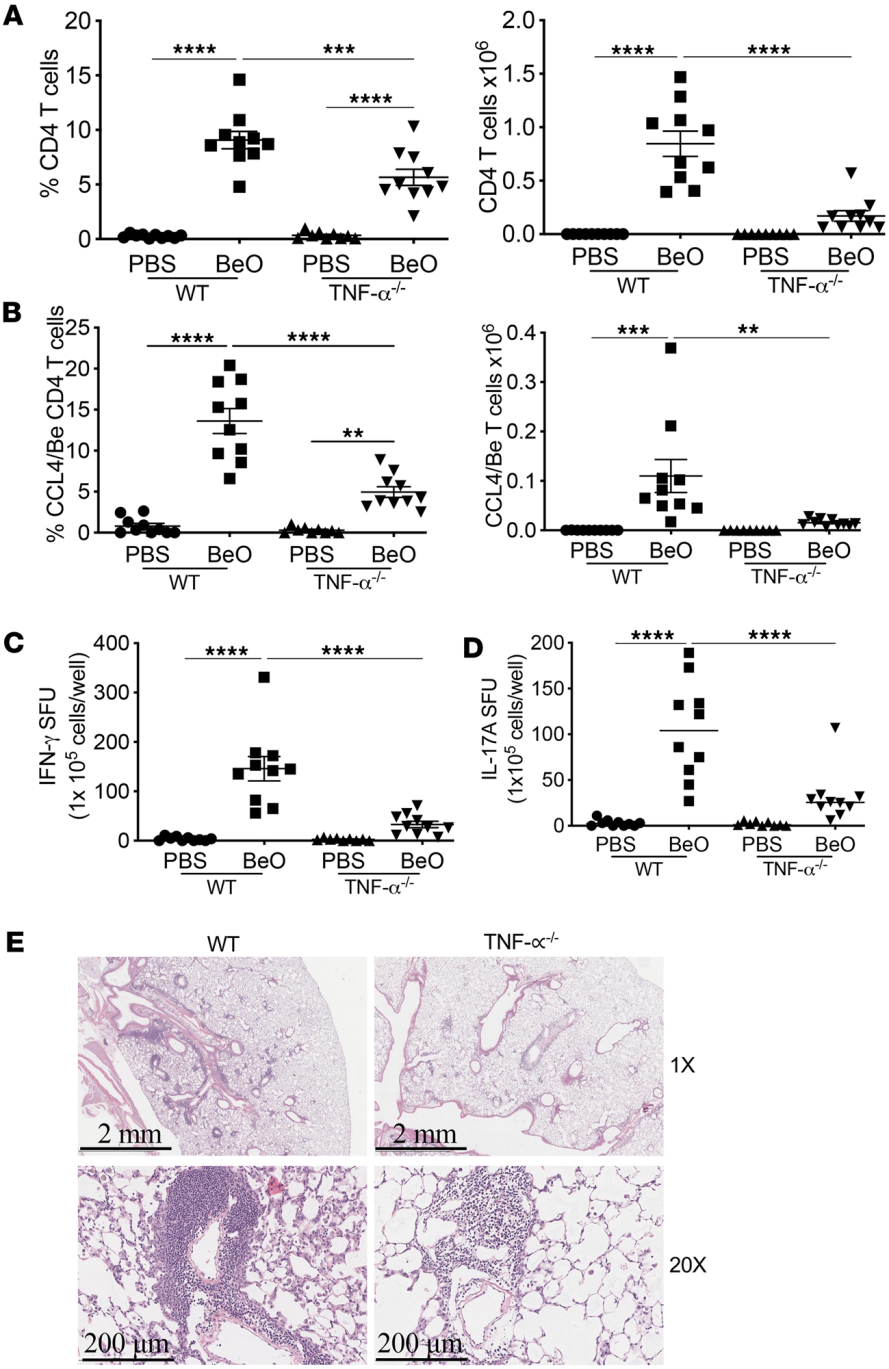
Reduced total and antigen-specific CD4^+^ T cells in BeO-exposed TNF-α–deficient HLA-DP2 Tg mice. HLA-DP2 Tg WT and TNF-α^–/–^ mice were intratracheally exposed to PBS or BeO/LPS per the standard protocol. (**A** and **B**) Data plots show the frequency (left) and number (right) of tissue-resident (**A**) and HLA-DP2–CCL4/Be tetramer binding (**B**) CD4^+^ T cells. (**C** and **D**) Data plots show IFN-γ (**C**) and IL-17A (**D**) ELISPOT responses as spot-forming units (SFUs) per 100,000 purified CD4^+^ T cells stimulated with BeSO_4_ (100 μM). Each data point represents an individual mouse; values are shown as (mean ± SEM) from 2 combined independent experiments. (**E**) Low (1X) and high (20X) magnification of H&E-stained lung sections from BeO/LPS-exposed WT and TNF-α^–/–^ mice. One-way ANOVA was used to determine statistical significance. **P < 0.01, ***P < 0.001, ****P < 0.0001.

TNF-α induces leukocyte secretion of chemokines, including CCL3 and CCL4, during inflammation and infection (33, 34). Our transcriptomics data show increased gene expression of TNF-α (Figure 6E) and significant upregulation of the TNF-α pathway among HLA-DP2 tetramer-binding T cells (Figure 7A). To directly test the involvement of this cytokine in the development of Be-modified chemokine-specific CD4^+^ T cell responses, we utilized HLA-DP2 Tg TNF-α^–/–^ mice. On day 21, the frequency and number of CD4^+^ T cells were significantly reduced in BeO-exposed TNF-α^–/–^ mice compared with the WT mice (Figure 8A). Similarly, HLA-DP2–CCL4/Be tetramer staining of lung cells revealed that mice deficient in TNF-α had a reduced frequency (~3-fold) and number (~7-fold) of antigen-specific CD4^+^ T cells compared with mice with functional TNF-α (Figure 8B). Next, we assessed IFN-γ and IL-17A CD4^+^ T cell responses in BeO/LPS-exposed lungs, since both cytokines contribute to granulomatous inflammation (35). BeO exposure resulted in more than a 2-fold reduction in the number of IFN-γ and IL-17A–producing CD4^+^ T cells in TNF-α^–/–^ mice compared with WT mice (Figure 8, C and D). Finally, histology of lung specimens showed that TNF-α deficiency also reduced Be-associated cellular aggregates in the lungs (Figure 8E). Thus, TNF-α enhances the Be-specific immune response, as evidenced by the reduction of CCL4/Be-specific and cytokine-producing cells in its absence.

### CD4^+^ T cell cross-reactivity to CCL/Be ligands in the lungs of Be-exposed HLA-DP2 Tg mice.

In CBD, a set of CD4^+^ T cell clones bearing a related CDR3β TCR motif demonstrated cross-reactive responses to Be-dependent CCL3 and CCL4 peptides (7). Because these chemokine epitopes only differ at 3 positions and the amino acid differences are conserved, this observation was not unexpected. A high degree of similarity also exists between mouse and human chemokine sequences. To determine if similar cross-reactivity to the orthologous murine CCL3/4 peptides is present in our animal model of CBD, we costained lung-resident CD4^+^ T cells from BeO/LPS-exposed HLA-DP2 Tg mice with both HLA-DP2–CCL/Be tetramers, conjugated to different fluorochromes. Single tetramer staining showed that approximately 10% of lung CD4^+^ T cells stained with either the HLA-DP2–CCL4/Be or the HLA-DP2–CCL3/Be tetramers (Supplemental Figure 7A). However, costaining revealed a large fraction of T cells binding both tetramers, and closer examination showed distinct CD4^+^ T cell clusters that varied among individual mice and were indicative of their relative binding avidities to both ligands (Supplemental Figure 7, A–C). The overall percentage of T cells that were cross-reactive to the Be-dependent CCL epitopes (i.e., bound to both tetramers) was 47.2% with a large observed range (Supplemental Figure 7C; range, 20%–65%). In summary, these findings indicate that Be^2+^ exposure produces a similar magnitude of CCL3- or CCL4-specific CD4^+^ T cell responses in the lungs of exposed mice.

### HLA-DP2–CCL4/Be-CAR-T cells limit the in vivo accumulation of chemokine-specific CD4^+^ T cells in the lungs of BeO-exposed mice.

**Figure 9:**
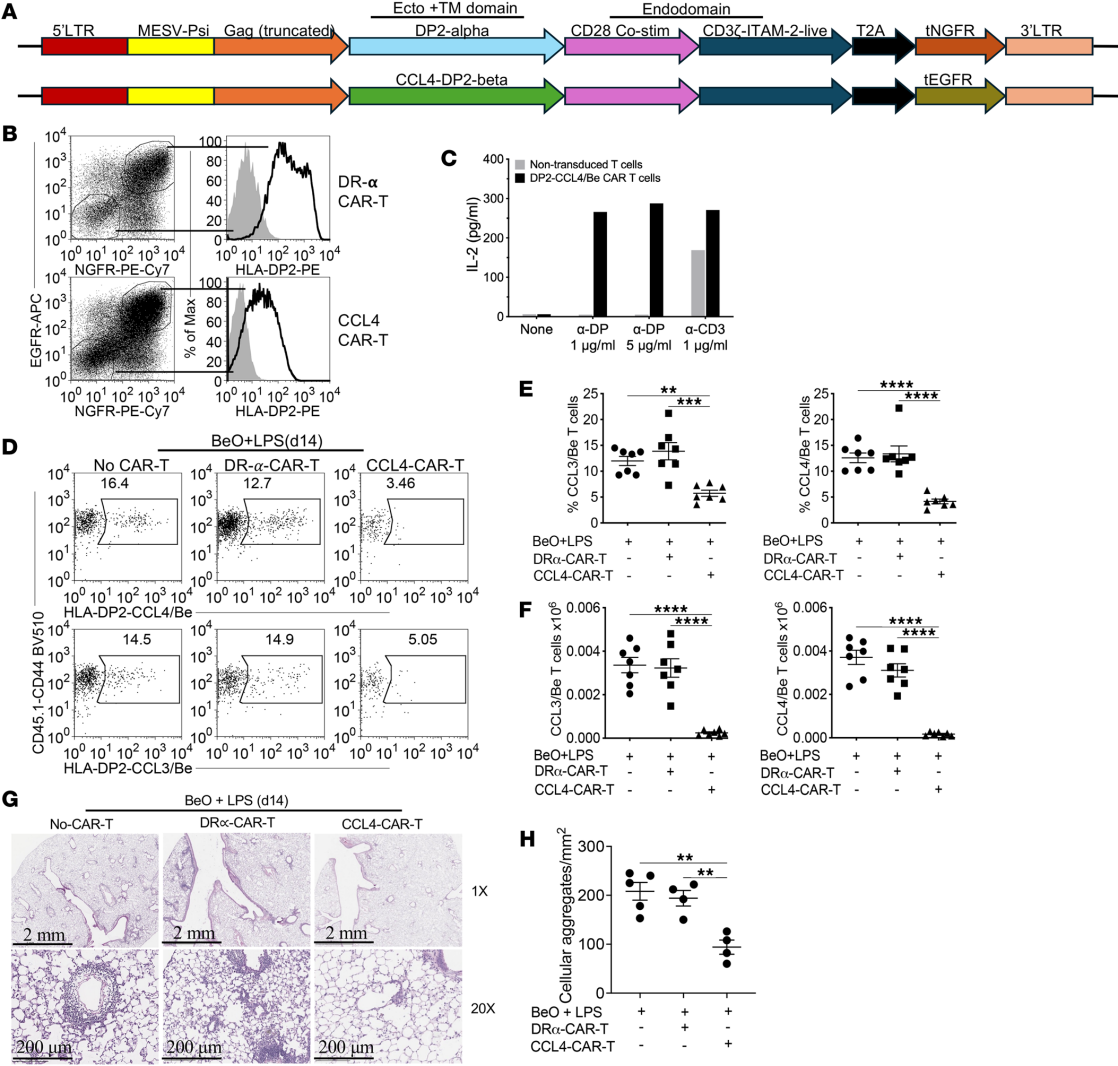
Reduced frequency of CCL/Be-specific CD4^+^ T cells in the lungs of BeO-exposed mice treated with HLA-DP2/CCL4-CAR-T cells. (**A**) Organization of MSCV vectors shows CARs expressing ecto- and transmembrane domains of the HLA-DP2 α-chain (top) and the β-chain (bottom), both connected to CD28/CD3ζ-ITAM-2-live. Peptides (derived from DRα or CCL4) are covalently attached to the N-terminus of β-chain by a linker. (**B**) Flow cytometry plots show representative transduction efficiencies (left, double positive gated population) and HLA-DP2 CAR expression (right) with either the DRα-peptide (top) or CCL4 peptide (bottom) on day 4 post-transduction. (**C**) IL-2 ELISA shows that the HLA-DP2–CCL4/Be CAR expressed on CD3^+^ mouse T cells can signal transduce and produce IL-2 in response to plate-bound mAb. (**D**) Representative HLA-DP2–CCL4/Be (top) and HLA-DP2–CCL3/Be tetramer staining of lung-resident CD4^+^ T cells in HLA-DP2 Tg mice exposed to BeO/LPS and treated with and without CAR-T cells. (**E** and **F**) Frequency (top, **E**) and number (bottom, **F**) of CCL3/Be- and CCL34/Be-specific CD4^+^ T cells in the lungs of BeO-exposed mice treated with CAR-T cells. (**G**) H&E staining showing low (1X) and high (20X) magnification of lung tissues and quantification of cellular aggregates (**H**) in BeO-exposed lungs, either untreated or treated with control CAR-T cells (DRα-CAR-T) or CCL4 CAR-T cells (CCL4-CAR-T) on day 8 and examined on day 21. Data are shown as mean ± SEM, pooled from 2 independent experiments. One-way ANOVA was used to determine statistical significance. **P < 0.01, ****P < 0.0001.

Corticosteroids are the standard treatment for CBD, but this therapy is nonspecific and associated with several risks, including immunosuppression, infections, and other steroid-related effects. CAR-T cells have recently proven effective in treating various forms of cancers and is an emerging area of investigation in autoimmune diseases (36–40). Here, we designed second-generation CAR-T cells containing a CD28/CD3ζ endodomain with an active middle ITAM motif connected to the transmembrane domains of both HLA-DP2 chains. The ectodomain of the HLA-DP2 β-chain also contained covalently attached CCL4 or control peptide (Figure 9A). Given the extensive cross-reactivity of T cells to the CCL3 and CCL4 peptides (Supplemental Figure 7), we predicted the CAR-T cells would effectively eliminate most T cells with antigen specificity to either of these epitopes. Negatively enriched CD3^+^ T cells from CD45.1 congenic mice were activated overnight in culture and transduced with retroviruses separately encoding the HLA-DP2 α-chain marked by EGFR and the β-chain with NGFR. Transduction efficiencies, defined as T cells expressing both EGFR and NGFR, were routinely 50%–70% of total CD3^+^ T cells (Figure 9B). In vitro stimulation of CAR-T cells with plate-bound mAb confirmed the ability of the transduced T cells to signal through their CARs and secrete IL-2 (Figure 9C). To assess the efficacy of HLA-DP2 CAR-T cells, BeO/LPS-exposed HLA-DP2 Tg mice were adoptively transferred with 6 × 10^6^ DP2-CCL4/Be-CAR-T cells or DP2-DRα-CAR-T cells on day 8 after briefly exposing the DP2-CCL4/Be-CAR-T cells to BeSO_4_ (50 mM) to generate the ligand for cognate TCRs. Mice were sacrificed on day 21, and HLA-DP2 tetramer staining of individual nontreated and CAR-T cell–treated mice is shown in Figure 9D. Compared with the nontreated BeO/LPS-exposed group, mice adoptively transferred with CCL4/Be-CAR-T cells showed a significant reduction in the frequency and number of lung-resident CD4^+^ T cells (Supplemental Figure 8) and CD4^+^ T cells binding the HLA-DP2–CCL4/Be (Figure 9E) and HLA-DP2–CCL3/Be (Figure 9F) tetramers. Transfer of nonspecific DP2-DRα-CAR-T cells had no affect on these antigen-specific cell populations (Figure 9, D and F). Importantly, H&E staining of lung tissues showed a significant reduction in inflammation, determined by the presence of cellular aggregates, in BeO-exposed HLA-DP2 Tg mice treated with CCL4/Be-CAR-T cells compared with BeO-exposed control groups treated with no CAR-T cells or nondisease-specific DP2-DRα-CAR-T cells (Figure 9, G and H). These data demonstrate that CCL4/Be-CAR-T cells effectively reduce CCL/Be-specific CD4^+^ T cells in the lungs of BeO-exposed mice.

## Discussion

While the mechanisms of CBD pathogenesis involve participation from innate and adaptive immune cells, much remains unknown about how these arms of the immune system interact to cause disease. Here, we demonstrate that Be exposure resulted in the accumulation of antigen-specific CD4^+^ T cell subsets in the lung parenchyma, and this response was dependent on the chemokines CCL3 and CCL4. HLA-DP2 Tg CCL3^–/–^ (CCL3^–/–^) mice showed reduced total and antigen-specific CD4^+^ T cells in the lungs. Furthermore, CCL3^–/–^ mice, which also lack CCL4, exhibited fewer cellular aggregates, suggesting reduced lung inflammation due to the diminished presence of pathogenic CD4^+^ T cells. Thus, the current study unravels the importance of chemokines in recruiting and expanding pathogenic CD4^+^ T cells to the lungs in response to BeO exposure (Figures 4 and 5) and confirms that these chemokines are the source of dominant Be-complexed neoantigenic CD4^+^ T cell epitopes. Additionally, this study highlights the importance of the hematopoietic compartment, especially DCs, and TNF-α signaling in controlling chemokine-specific T cell responses.

A key factor driving CBD pathogenesis is the extent of Be exposure in genetically susceptible individuals within the lungs. In the United States, the Occupational Safety and Health Administration (OSHA) has implemented measures to minimize occupational exposure to Be and its compounds in the workplace. Despite these regulatory efforts, however, workers in Be processing industries worldwide remain at continued risk of inhaling Be^2+^ particles, underscoring the importance of understanding the effects of Be interaction with the immune system. Structural studies of HLA-DP2 bound to a T cell–stimulating mimotope revealed that βGlu69 participates in tetrahedral coordination of the Be^2+^ cation within an acidic P4 pocket (41). Be binding alters both the charge and conformation of peptides bound in the HLA-DP2 groove, reshaping the topology of the HLA-DP2–self-peptide complex to form neoantigens (41). This capacity to present Be-modified self-peptides explains the genetic linkage between bGlu69-expressing HLA-DP alleles and CBD.

Inflammation plays a critical role in the pathogenesis of granulomatous diseases such as CBD and is driven in part by chemokines. These low–molecular weight molecules are secreted by both hematopoietic and nonhematopoietic cells, including mononuclear phagocytes and epithelial cells. Chemokines act as homeostatic or inflammatory mediators and are essential for directing leukocyte migration (42) as well as regulating cellular proliferation, oxidative burst, and tissue repair (42). In pulmonary diseases such as sarcoidosis, idiopathic pulmonary fibrosis, and hypersensitivity pneumonitis, chemokines contribute to alveolitis and granuloma formation (43, 44). Among inflammatory C-C motif chemokines, CCL3 (MIP-1α) and CCL4 (MIP-1β) are highly homologous and show marked upregulation in response to infection and sterile-particle exposure (7, 29, 45). In the present study, we generated HLA-DP2 Tg mice genetically deficient in CCL3 and hypomorphic for CCL4 to dissect the contributions of these chemokines in the innate and adaptive immune responses during CBD pathogenesis. Both WT and CCL3^–/–^ HLA-DP2 Tg mice had similar numbers of lung macrophages and DCs following PBS or BeO exposure (Figure 1), and costimulatory molecule expression on pulmonary DC subsets further indicated that CCL3 and CCL4 deficiency had no significant effect on innate immune cell activation (Figure 1D). However, BeO-exposed CCL3^–/–^ mice exhibited a significant reduction in the accumulation of CD4^+^ T cells in the lungs at day 21. We interpret this reduction as reflecting the decreased availability of CCL3 and CCL4 as sources of Be-modified neoantigens, rather than an effect on CD4^+^ T cell recruitment. Chemokine receptor 7 (CCR7), which mediates T cell migration to sites of injury or inflammation in response to chemokines, was expressed at similar levels on lung CD4^+^ T cells from both BeO/LPS-exposed WT and CCL3^–/–^ mice. Moreover, in a non-Be exposure model, WT and CCL3^–/–^ mice exposed to *A*. *nidulans* showed comparable frequencies and numbers of lung CD4^+^ T cells. Thus, our data highlight that CCL3 and CCL4 deficiency does not influence CD4^+^ T cell migration and support the conclusion that the reduction in lung CD4^+^ T cells in CCL3^–/–^ mice is driven primarily by the loss of antigen-specific responses rather than altered homing.

Since DCs are professional APCs that prime and activate T cells, the reduced CCL/Be-specific T cell response observed in mice lacking conventional DCs demonstrates their role in maintaining these responses in BeO-exposed lungs (Figure 4). This reduction likely reflects their function as primary APCs rather than their contribution as a source of chemokines, given that other cells in the lungs, such as macrophages, appear to function normally. Furthermore, bone marrow chimera experiments, in which the hematopoietic compartment of CCL3^–/–^ mice was replaced with WT cells, further implicate hematopoietic-derived DCs in CBD pathogenesis. Thus, our data consistently demonstrate that HLA-DP2 Tg mice deficient in CCL3 and CCL4 require DCs for the induction of CCL/Be-specific CD4^+^ T cell responses.

Recent studies have advanced our understanding of the self-peptides that complete the Be-dependent ligand for antigen-specific CD4^+^ T cells in CBD. Utilizing decapeptide positional scanning libraries and T cell hybridomas expressing selected αβTCRs from Be-specific, lung-derived CD4^+^ T cell clones, peptides derived from CCL3 and CCL4 activated the hybridomas when presented by HLA-DP2–expressing cells in the presence of Be. In the BAL of patients with active disease, HLA-DP2–CCL/Be tetramer staining confirmed that a substantial fraction of CD4^+^ T cells were specific to these ligands, establishing them as dominant antigens in CBD. Follow-up studies in Be-exposed HLA-DP2 Tg mice also identified lung-resident CD4^+^ T cells sharing these specificities, underscoring the relevance of this animal model to human disease (7). Further examination revealed that the large majority of these antigen-specific CD4^+^ T cells were primarily Th1 and Treg cells (8). Similarly, in a related disease model, CD4^+^ T cells recruited to the lungs of mice exposed to silica complexes were comprised of mixed populations of Th1 and Tregs (46–48). To delve further into their phenotypes, we investigated the transcriptional profile of CD4^+^ T cells isolated from the lungs of Be-exposed mice and flow-sorted based on their expression of the activation marker CD44 and HLA-DP2–CCL4/Be tetramer binding. Antigen-specific T cells (Tet^+^) and CD44^+^ Tet^–^ T cells were comprised mostly of CD4^+^ Th1, Th17, and Tregs (Figure 6E). The significant overlap observed in the Tet^+^ and Tet^–^ T cells may be attributable to T cells within the Tet^–^ fraction that recognize other Be-associated epitopes, including CCL3, plexin-A4 (7, 49). Pathway enrichment analysis revealed significant upregulation of the IL-2 Stat-5, mTORC1, and TNF-α signaling pathways, suggesting that multiple subsets of CCL/Be-specific CD4^+^ T cells contribute to the inflammation seen in Be-induced disease.

Corticosteroids are the mainstay of therapy for CBD, but significant side effects often limit their long-term use (50). We sought to design an immunotherapy that allows specific targeting of CCL/Be-specific CD4^+^ T cell populations while leaving the broader CD4^+^ T cell compartment intact in our murine model of CBD. To that end, we developed a potentially novel peptide-MHC class II (pMHCII) CAR that featured HLA-DP2 extracellular and transmembrane domains with the CCL4 peptide covalently attached to the β-chain of the HLA-DP2 molecule. To generate the appropriate T cell ligand, transduced CAR-T cells were incubated in Be-containing medium before use. The CAR construct also incorporated second-generation CD28/CD3ζ signaling domains connected to both HLA-DP2 chains, with each CD3ζ cytoplasmic tail containing a single functional immunoreceptor tyrosine-based activation motif (ITAM). This design was chosen to reduce CAR-T cell exhaustion, limit inflammation triggered by CAR-T cell activation, and enhance cell persistence by minimizing activation-induced cell death (51). The novelty of this approach lies in depleting only CD4^+^ T cells reactive to specific epitopes from CCL3 and CCL4. Other versions of pMHCII CARs have been used to target pathogenic CD4^+^ T cells in models of experimental autoimmune encephalitis (EAE), collagen-induced autoimmune arthritis, and type 1 diabetes (21, 23, 38).

We tested this cellular therapy in our HLA-DP2 Tg animal model of CBD, which offers several advantages for evaluating CAR-T cell efficacy. It does not require an adoptive transfer of TCR Tg T cells or clonally expanded pathogenic CD4^+^ T cells. Instead, the model depends on exposure to an environmental agent. Inhalation of Be-containing compounds represents a real-world exposure that naturally elicits both innate and adaptive immune responses in mice that includes a complex pathogenic CD4^+^ T cell response to multiple HLA-DP2–restricted Be-modified antigens. Thus, therapeutic CAR-T cells must target heterogeneous responding CD4^+^ T cell populations that arise in vivo and reflect a diversity of avidities for their ligands. Here, we demonstrated that CCL4/Be-epitope expressing pMHCII CARs eliminated a large percentage of CCL/Be-specific CD4^+^ T cells within the lungs of Be-exposed mice and reduced the number and size of cellular aggregates that form in the lungs with Be exposure.

In contrast to prior animal studies that employed only CD8^+^ CAR-T cells to invoke a cytolytic mechanism of activity, our approach utilized both CD4^+^ and CD8^+^ CAR-T cells, and future studies can dissect their relative contributions to therapeutic efficacy. In our humanized animal model of CBD, the number and frequency of CCL4/Be- and CCL3/Be-specific CD4^+^ T cells were reduced to a similar extent after CAR-T cell treatment, reflecting the high degree of T cell cross-reactivity to both ligands. Additionally, the number of cellular aggregates was significantly reduced in the lungs of Be-exposed mice treated with CAR-T cells, underscoring the therapeutic efficacy of ligand-specific CAR-T cells. While other antigens are targeted by autoreactive CD4^+^ T cells in CBD (7, 49), our evidence suggests that control of T cells specific to immunodominant epitopes can mitigate the broader pathologic immune response. Thus, we have developed a powerful experimental tool to elucidate the roles of distinct antigen-specific CD4^+^ T cells in CBD. Furthermore, these findings are consistent with other peptide-MHC class II CAR formats, which have demonstrated success in mouse models of T cell–mediated autoimmune disorders such as autoimmune arthritis and type 1 diabetes (21, 52). Taken together, pMHCII CAR technology is positioned as a promising next-generation precision cellular therapy platform that could eventually advance toward preclinical trials as a treatment regimen in refractory CBD and other T cell–mediated autoimmune diseases.

In summary, this study highlights the role of chemokines as major determinants in the progression of CBD, establishes the role of DCs and TNF signaling in regulating an optimal chemokine/Be-specific CD4^+^ T cell response, and, finally, develops a pMHCII-CAR-T cell therapy as an alternative to the long-standing corticosteroid treatment recommended for patients with CBD.

## Methods

### Sex as a biological variable.

Both male and female mice were included in the study, and similar findings were observed in both sexes.

### Mice and treatment.

WT HLA-DP2 Tg C57BL/6 and HLA-DP2 Tg FVB/N mice were bred and maintained in our animal facility. HLA-DP2 Tg CCL3^–/–^ and Zbtb46-DTR mice were generated by crossing WT mice with B6.129P2-Ccl3tm1Unc/J (CCL3^–/–^) and B6(Cg)-*Zbtb46^tm1(HBEGF)Mnz^*/J mice, both obtained from Jackson Laboratories. For bone marrow chimera experiments, B6.SJL-PtprcaPep3b/BoyJ (CD45.1 C57BL/6) mice (Jackson Laboratories) were crossed to WT mice to generate CD45.1 congenic HLA-DP2 Tg mice. To model CBD, 6- to 8-week-old mice were lightly anesthetized with isoflurane and exposed to 50 μL of sterile PBS or PBS containing 100 μg of endotoxin-free BeO (NIST, designated standard reference material 1877) on days 0, 1, 2, 14, 15, 18, and 19 and treated with a single dose (10 μg) of LPS on day 14 via oropharyngeal aspiration as previously described (48). Mice were euthanized on day 21. To examine the early effects of Be-exposure, HLA-DP2 Tg mice were exposed to endotoxin-free BeO on days 0, 1, 2, and a single dose of LPS on day 8. Mice were euthanized on day 12 for analysis.

### A.

*nidulans culture and infection*. *Aspergillus nidulans* (Eidam) Winter MYA-3632, AN1 strain was obtained from the American Type Culture Collection (ATCC). Fungi were cultured on potato dextrose agar plates at 37°C and harvested on day 8. Spores were prepared in phosphate-buffered saline (PBS, pH 7.4), counted, and resuspended at 2 × 10^9^ spores/mL in PBS. WT and CCL3^–/–^ mice were lightly anesthetized and exposed to 50 μL of the spore suspension via oropharyngeal aspiration on day 0. Mice were euthanized on day 14 for lung CD4^+^ T cell analysis.

### Bone marrow chimera mice.

Eight-week-old HLA-DP2 Tg CD45.1 C57BL/6, WT, or CCL3^–/–^ mice were irradiated with 500 rads twice at a 6-hour interval using an x-ray irradiator. One hour after the last irradiation, HLA-DP2 Tg WT, and CCL3^–/–^ mice (CD45.2) were reconstituted with bone marrow from HLA-DP2 Tg C57BL/6 CD45.1 mice to investigate the role of the hematopoietic compartment in CBD. To study the role of DCs in CBD, HLA-DP2 Tg CD45.1 C57BL/6 mice were reconstituted i.v. with 2 × 10^6^ bone marrow (BM) cells from HLA-DP2–ZbTb46-DTR Tg mice. Reconstituted mice received enrofloxacin (Baytril 100) in drinking water for 6 weeks. At 8 weeks after reconstitution, chimerism (CD45.1 versus CD45.2 expression) was assessed by flow cytometry before mice were used for experiments.

### Preparation of lung cells.

Mice were anesthetized, and 2 minutes before euthanasia, received a retro-orbital injection of CD45-APC-Cy7 mAb (5 μg/mouse; clone 30-F11, BioLegend), to distinguish circulating cells from tissue-resident cells (8, 52). The lungs were perfused with ice-cold PBS, removed, minced, and digested for 30 minutes in complete culture media (RPMI-10) containing 1 mg/mL collagenase D (Sigma-Aldrich). After digestion, the lung tissue was mechanically disrupted using 16G and 18G needles. Collagenase activity was quenched with cold PBS, and lung cells were pelleted by centrifugation at 300 × *g* for 5 minutes. Finally, the cell suspension was filtered through a 100 μM cell strainer and resuspended in RPMI-10.

### Flow cytometry and intracellular staining.

Lung cells were stained with Fc receptor-blocking mAb, and fluorescently conjugated mAbs to cell surface antigens were diluted in PBS containing 1% FBS, 0.05% sodium azide, and 0.5 μg/mL CD16/CD32 (Tonbo; clone 2.4G2). Cells were incubated for 30 minutes at 4°C, washed, and resuspended in FACS buffer. The following antibodies were used for multi-parameter FACS analysis: CD3 (Tonbo; 17A2), CD44 (Tonbo, IM7), CD4 (RM4-5, RM4-4), CD8 (53-6.7), CD69 (H1.2F3), B220 (RA3-682), CD103 (2E7), Ki67 (3E12), PD-1 (RMP1-30), CD62L (MEL-14), HLA-DP (Tü39), and HLA-DP (purified from hybridoma, B7.21), all purchased from BioLegend. To exclude innate cells (dump gate) in the analysis of tissue-resident CD4^+^ T cells, we utilized antibodies directed against CD11c (BD Biosciences, N418), CD11b (M1/70, ThermoFisher Scientific), IA/IE (M5.114.15.2, ThermoFisher Scientific), and Ly6G (1A8, BioLegend), purchased from BD Biosciences, Thermo Fisher Scientific, or BioLegend. Following extracellular staining, cells were fixed using a Foxp3/transcription factor staining buffer set (Thermo Fisher Scientific) for 30 minutes. Intracellular staining was performed at room temperature for 30 minutes using anti-CCL3 (DNT3CC), FoxP3 (FJK-16s), IFN-γ (XMG1.2), TNF-α (MP6-XT22), and IL-17A (TC11-18H10.1) monoclonal antibodies. Cells were resuspended in 1X Perm buffer and analyzed on either a BD FACSCanto II or BD Fortessa flow cytometer, with data analyzed using FlowJo software (v9.9.6).

### Tetramer staining.

An aliquot of lung cells from BeO/LPS-exposed mice was incubated with HLA-DP2–CCL3/Be and HLA-DP2–CCL4/Be tetramers (both at 20 μg/mL), conjugated to allophycocyanin (APC) or phycoerythrin (PE), respectively. Tetramers with covalent peptides were provided by the NIH Tetramer Core Facility at Emory University (Atlanta, GA) and incubated with BeSO_4_ (100 μM) for 30 minutes at 37°C before use. After 2 hours at 37°C, cells were washed and stained with surface antibodies as described above. Newly obtained lots of tetramers were validated before experimental use by staining T cell hybridomas expressing T cell receptors of known specificity or primary CD4^+^ T cells obtained from Be^2+^ exposed WT mice.

### qPCR.

Snap-frozen lung tissue samples were homogenized, and RNA was isolated using TRIzol (Invitrogen) and a mini-RNA isolation kit (Qiagen). Complementary DNA was synthesized using iScript Reverse Transcription Supermix (Bio-Rad). qPCR was performed with SYBR Green PCR Master Mix (Bio-Rad) on a QuantStudio 5 Real-Time PCR system (Applied Biosystems). Primers used for target genes were: mCCL3-forward (mCCL3-F): tgagagtcttggaggcagcga, mCCL3-reverse (mCCL3-R): TGTGGGTACTTGGCAGCAAACA, mCCL4-F: aacaacatgaagctctgcgt, mCCL4-R: AGAAACAGCAGGAAGTGGGA, β-actin-F: GACAGCTACGTGGGTGACGAA, β-actin-R: TTTTCCATGTCGTCCCAGTTG. The comparative Ct values for the genes of interest were normalized to the Ct value of β-actin. Gene expression fold changes relative to the control were calculated using the 2-ΔΔct method.

### Preparation of spleen cells for irradiation.

Spleens were harvested from mice, processed through a 70 μM cell strainer, and rinsed with PBS. Cells were pelleted by centrifugation at 300 × *g* for 5 minutes. After RBC lysis, splenocytes were filtered through a 70 μM cell strainer and resuspended in RPMI-10. Splenocytes were then exposed to 3 Gy of ionizing radiation using a MultiRad350 X-ray irradiator (Precision X-ray). After irradiation, cells were centrifuged, washed, and resuspended in RPMI-10.

### Analysis of IFN-γ and IL-17A secretion by CD4^+^ T cells using ELISPOT assay.

ELISPOT plates (ImmunoSpot M200, BD Biosciences) were coated with anti-IFN-γ or anti-IL-17A capture mAbs (MAbtech) overnight and blocked with RPMI-10 for 2 hours at room temperature (48). Lung CD4^+^ T cells were positively selected using magnetic beads, and 1 × 10^5^ CD4^+^ T cells were combined with 4 × 10^5^ irradiated spleen cells from an unexposed HLA-DP2 Tg mouse in duplicate. Cells were incubated overnight at 37°C in medium or 100 μM BeSO_4_. IFN-γ or IL-17A detection mAbs (MAbtech) were added, and spots were visualized using avidin-horseradish peroxidase and 3-amino-9-ethylcarbazole substrate (BD Biosciences). ELISPOT plates were analyzed using a CTL Immunospot Analyzer (Cellular Technology Ltd), and results were reported as mean ± SEM spot-forming units (SFUs) per well minus background SFUs (medium alone).

### Histology.

Lungs were inflated with 0.5 mL of 1% agarose solution containing 4% PFA, then further fixed in 10% neutral-buffered formalin for 24 hours and stored in 70% ethanol for histopathological analysis. H&E staining was performed using Histowiz. To quantify mononuclear cell infiltrates, whole slide imaging was performed on H&E-stained lung sections from formalin-fixed, paraffin-embedded tissue. Pyramidal tiff files were analyzed using QuPath-v.0.5.1-arm64. Stain vector values were automatically determined, and cells were counted by adjusting the cell detection threshold to maximize the difference between areas containing perivascular mononuclear infiltrates from unaffected areas (29).

### Library construction and RNA-seq.

For transcriptomic analysis of CD4^+^ T cell subsets, lung cells from Be-exposed HLA-DP2 Tg mice were prepared, and CD4^+^ T cells were purified using the MojoSort Mouse CD4 T Cell Isolation Kit (BioLegend). Enriched CD4^+^ T cells were incubated with HLA-DP2–CCL4/Be tetramer for 2 hours at 37°C. Cells were stained with fluorescently labeled mAbs directed against CD4, CD44, and dump gate markers (CD8, B220, CD11c, CD11b, Ly6C) and FACS-sorted into 3 distinct CD4^+^ T cell populations based on CD44 expression and HLA-DP2–CCL4/Be tetramer staining: CD44^+^tetramer^+^ (Tet^+^), CD44^+^tetramer^–^ (Tet^–^), and CD44^–^tetramer^–^ (DN). Library construction and sequencing were conducted by the Genomics and Microarray Core Facility at the University of Colorado Anschutz Medical Campus. RNA quality and integrity were assessed using an Agilent Tape Station 2200, and libraries were constructed using the Illumina TruSeq mRNA library construction kit. Paired-end sequencing (125 cycles) was performed on an Illumina HiSeq 4000. RNA-seq data was aligned using STAR v2.7.10a with the GRCm39 genome and gencode vM29 annotation. Read quantification was performed with featureCounts v2.0.4 using the parameters “-s 2 -p-countReadPairs” DESeq2 v1.38.3 in R was used for differential expression analysis. Genes with an absolute log_2_(fold change) ≥ 1 and an *P*_adj_ < 0.05 were used for over-representation analysis using the R package ClusterProfiler v4.12.0 with the enricher function. The hallmark and C5 ontology mouse gene set from the Molecular Signatures Database (MsigDB) were used to identify statistically significant gene sets (*P*_adj_ < 0.05). ClusterProfiler was also used to generate enrichment dot plots. The gene ratio was calculated as the number of overlapping genes within a specific pathway divided by the total number of overlapping genes in the entire signature set.

### CAR-T cell design and preparation.

CARs were constructed using gBlocks (IDT) encoding the extracellular domains of the HLA-DP2 α- and β-chains, with CCL4 or DRα peptide sequences connected to the N-terminus of the β-chain by a 14 amino acid linker. These constructs were introduced into separate pMSCV vectors by Gibson Assembly (New England Biolabs). The vectors include a CD28/CD3z endodomain (second generation) with a middle active ITAM motif (ITAM2 live) and T2 self-cleaving peptides that connect NGFR to the α-chain and EGFR to the β-chain to track transduction efficiencies. CD3^+^ T cells from C57BL/6 CD45.1 congenic mice were transduced to distinguish adoptively transferred CAR-T cells from recipient T cells for tracking homing and persistence. In brief, splenic CD3^+^ T cells were negatively enriched using an EasySep mouse T cell isolation kit (STEMCELL Technologies) and stimulated overnight with anti-CD3/CD28 beads (Gibco) at a 1:3 cell-to-bead ratio in the presence of rIL-2 (40 U/mL; STEMCELL Technologies) and rIL-7 (10 ng/mL; R&D Systems). For transduction, prepared retroviral supernatants and activated T cells were added to retronectin-coated (Takara Bio) nontissue culture-treated 6-well plates and spinfected at 2,000*g* for 2.5 hours at 37°C. Cells were placed in a culture with cytokines, and the anti-CD3/CD28 beads were magnetically removed after 24 hours (STEMCELL Technologies). At 48 hours, transduction efficiencies were determined by flow cytometry using fluorescently labeled mAbs directed to EGFR (AY13), NGFR (ME20.4), HLA-DP (Tü39), and CAR-T cells were exposed to BeSO_4_ (50 μM) for 2 hours to generate the proper T cell epitope. For adoptive transfer, 6 × 10^6^ CAR-T cells were injected intravenously through the tail vein into BeO-exposed congenic mice on day 8.

### ELISAs.

CCL3 and CCL4 protein levels in the BAL of PBS- and BeO-exposed mice were determined by sandwich ELISA using DuoSet matched antibody pairs (R&D Systems). To assess T cell activation, CAR-T cells (1 × 10^5^) were cultured overnight in a 96-well flat-bottom plate coated with media or anti-CD3 (1 μg/mL) or anti-HLA-DP (1 and 5 μg/mL) monoclonal antibody. IL-2 was measured in the culture supernatants by ELISA (Invitrogen) following the manufacturer’s guidelines. Absorbance readings at 450 nM were measured using a VMax microplate reader (Molecular Devices), and concentrations were calculated using SoftMax Pro software (Molecular Devices).

### Statistics.

A 2-way ANOVA and unpaired Student’s *t* test were used to determine the significance of differences between groups (Prism v10.3.0, GraphPad Software Inc.). Data were presented as mean ± SEM. *P < 0.05 considered statistically significant.

### Study approval.

All animal experiments complied with the NIH guidelines and received approval from the IACUC of Colorado Anschutz Medical Campus, which is accredited by the American Association for Accreditation of Laboratory Animal Care.

### Data availability.

All data generated in the article are included in the Supporting Data Values file, and RNA-seq data are available in the GEO under accession no. GSE325899.

## Author contributions

MTF, MR, PF, TJF, and SMA designed research. MTF, MR, CJN, RMT, JMG, FM, DGM, AKM, JCO, MY, APF, LAM, CAD, and SMA performed research. TMB, RF, and AD analyzed large sequencing data sets. KMA analyzed histology. MTF, MR, PF, and SMA wrote the manuscript.

## Conflict of interest

The authors have declared that no conflict of interest exists.

## Funding support

This work is the result of NIH funding, in whole or in part, and is subject to the NIH Public Access Policy. Through acceptance of this federal funding, the NIH has been given a right to make the work publicly available in PubMed Central.

- NIH grant HL152756 (to APF and SMA).
- NIH grant AI015614 (to CAD).
- University of Colorado School of Medicine Translational Research Scholars Program (to SMA).
- Ann Theodore Foundation Breakthrough Sarcoidosis Initiative grant (to SMA).

## Supplementary Material

Supplemental data

Supporting data values

## Version 1 06/09/2026

In-Press Preview

## Version 2 07/22/2026

Electronic publication
