# Supplementary material for: Reduced CCL/Be-specific CD4+ T cells in CCL3-deficient or peptide-MHCII CAR-T cell–treated mice
Source: JCI Insight. 2026 Jun 2;11(14):e195868. doi: 10.1172/jci.insight.195868 (PMC13461158; doi:10.1172/jci.insight.195868)
Supplement: Supplementary file 1 [file jciinsight-11-195868-s193.pdf]

Supplemental Data

Reduced CCL/Be-specific $CD4^+$ T cells in CCL3-deficient or peptide-MHC II CAR-T cell-treated mice

Michael T. Falta[1#], Masoom Raza[1#], Caley J. Nevienski[1], Tonya Brunetti[2], Rui Fu[3], Rebecca M. Tucker[1], Joseph M. Gaballa[1], Faiz Minhajuddin[1], Kibrom M. Alula[1], Alberto Dinarello[1], Douglas G. Mack[1], Allison K. Martin[1], Joseph Onyiah[1], Michael Yarnell[4,5], Prashanth Francis[4], Terry J. Fry[4,5], Lisa A. Maier[1,6], Andrew P. Fontenot[1], Charles A. Dinarello[1], Shaikh M. Atif[1]*

[1] Department of Medicine, University of Colorado Anschutz Medical Campus, Aurora, CO 80045, United States of America

[2] Department of Immunology and Microbiology, University of Colorado Anschutz Medical Campus, Aurora, CO 80045, United States of America

[3] New York Genome Center, New York, NY 10013, United States of America

[4] Department of Pediatrics, University of Colorado Anschutz Medical Campus, Aurora, CO 80045, United States of America

[5] Center for Cancer and Blood Disorders, Children's Hospital Colorado, Aurora, CO 80045, United States of America

[6] Department of Medicine, National Jewish Health, Denver, CO 80206, United States of America

# MTF and MR contributed equally to this work.

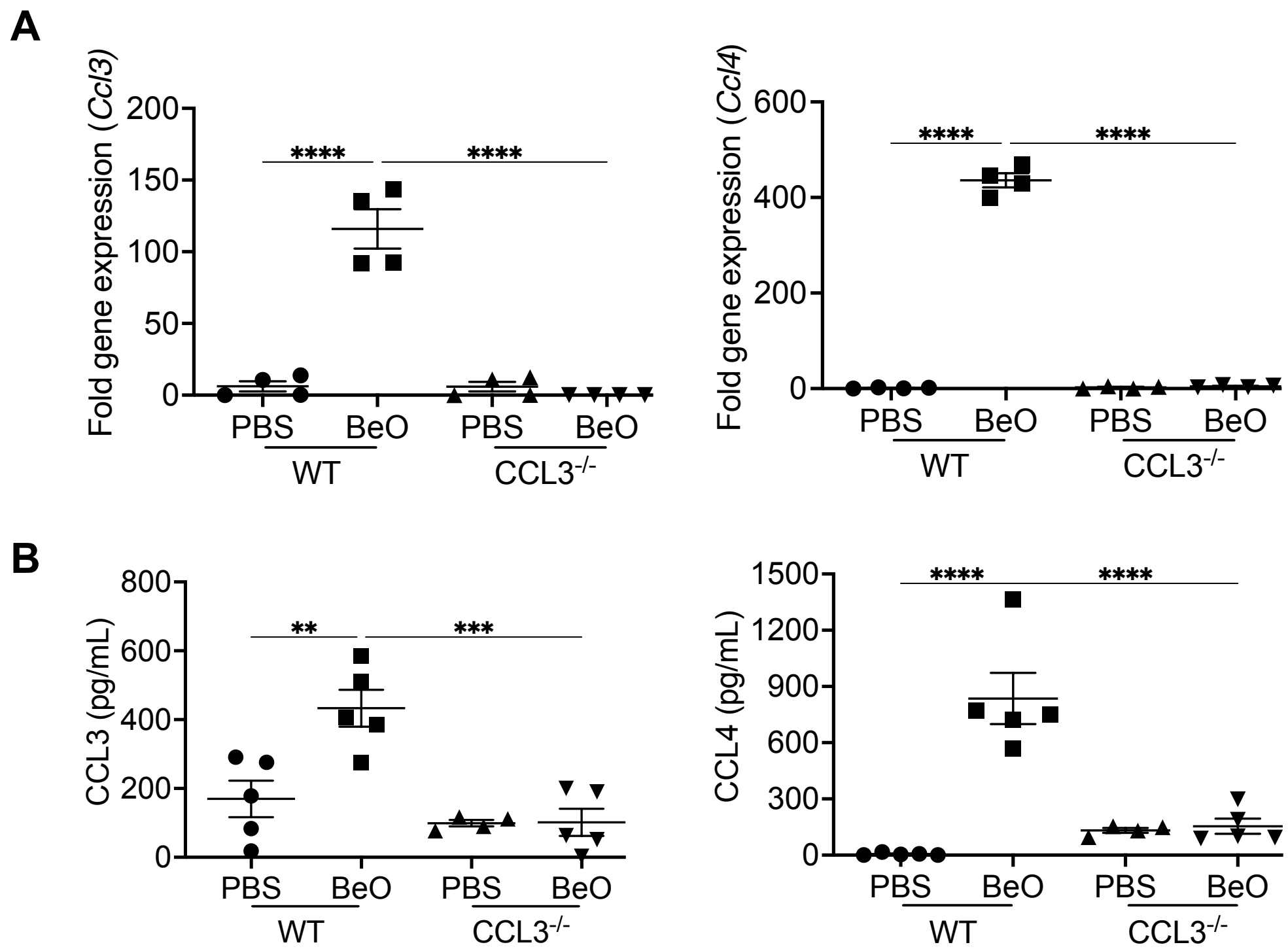

Supplementary Figure 1. Chemokine gene expression in the BAL fluid of BeO/LPS-treated HLA-DP2 Tg WT and CCL3$^{-/-}$ mice. Mice were intratracheally treated with 3 doses of BeO (100 µg) on days 0, 1, and 2 and a single dose of LPS (10 µg) on day 2. BAL was collected from individual mice on day 3. **(A)** Quantitative PCR (q-RT-PCR) shows fold gene expression of the *Ccl3* and C*cl4* genes relative to *Gapdh* in the lungs of PBS- or BeO/LPS-treated mice. **(B)** CCL3 and CCL4 protein levels were measured in the BAL fluid by ELISA. The data are representative of the (mean ± SEM) of two independent experiments. 1-way ANOVA was used to determine statistical significance. **P < 0.01, ***P < 0.001, ****P < 0.0001.

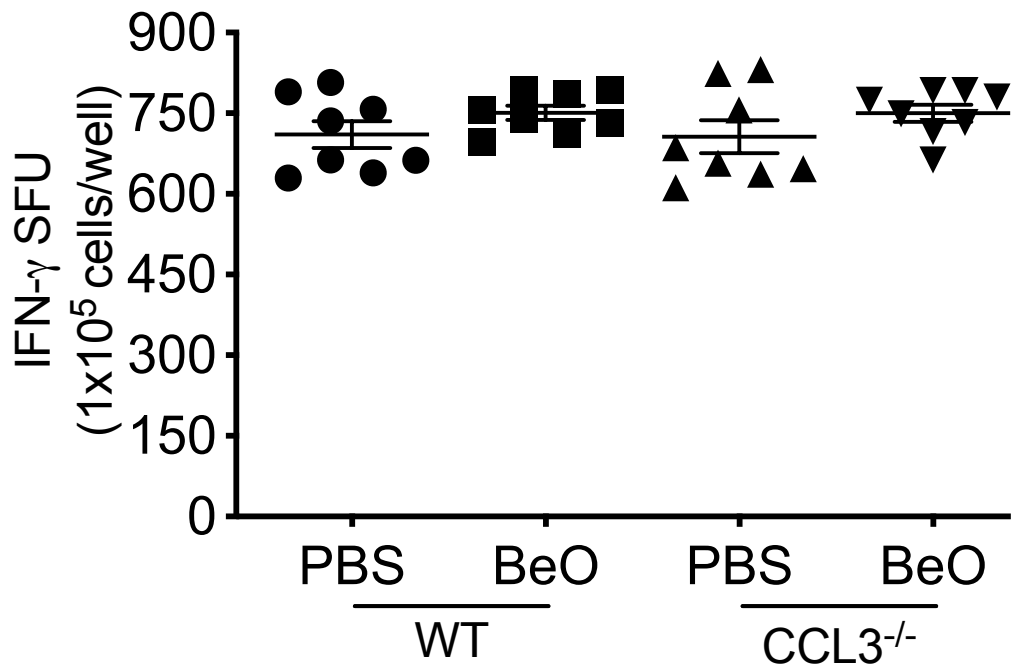

Supplementary Figure 2. Control ELISPOT assay. (**A**) HLA-DP2 Tg WT and CCL3$^{-/-}$ mice were exposed to PBS or BeO as described in the Materials and Methods. Lung CD4^{+} T cells were purified on day 21 and the treatment and control groups were stimulated *ex vivo* with phytohemagglutinin (1 mg/ml). The ELISPOT data plot shows T cell secretion of IFN-γ represented as spot-forming units (SFUs) per 100,000 purified CD4^{+} T cells stimulated overnight. Data represent the mean ± SEM across combined experiments.

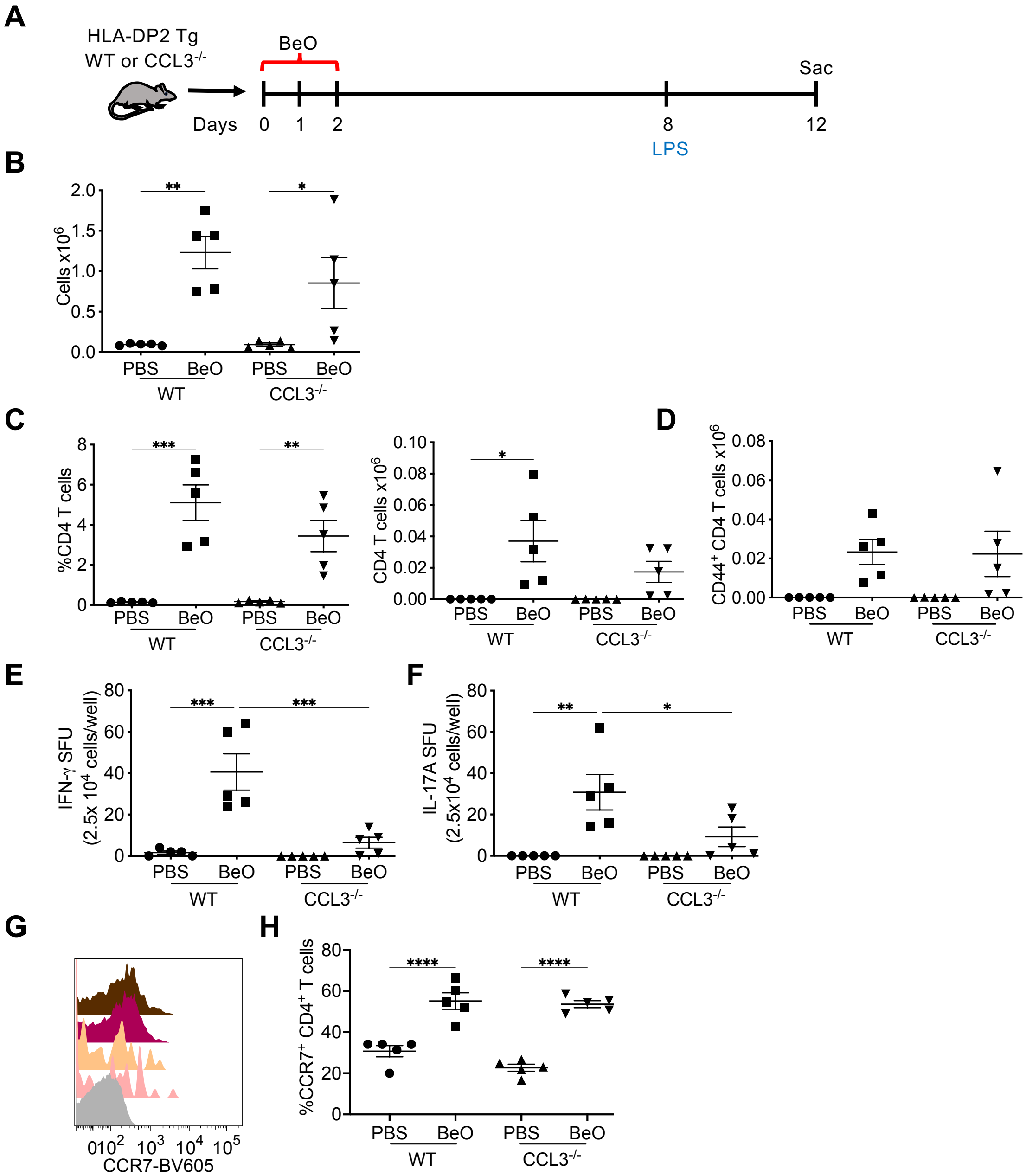

Supplementary Figure 3. IFN-γ and IL-17A-secreting cells are reduced in BeO-exposed CCL3$^{-/-}$ mice on day 12. HLA-DP2 Tg WT and CCL3$^{-/-}$ mice were exposed to BeO (100 µg) on days 0, 1, and 2 and treated with LPS (10 µg) on day 8. On day 12, mice were sacrificed 2 minutes after intravascular administration of anti-CD45 mAb. **(A)** Schematic design of the experiment. **(B)** Graph plots show the total number of cells in the lungs of PBS- or BeO/LPS-treated mice. **(C)** Frequency (left) and number (right) of CD4$^+$ T cells in the lungs were determined on day 21. (**D)** Number of activated (CD44$^+$) CD4$^+$ T cells. **(E-F)** ELISPOT responses for IFN-γ **(E)** and IL-17A **(F)** as spot-forming units (SFUs) per 25,000 purified CD4$^+$ T cells stimulated with $BeSO_4$ (100 µM). **(G)** Representative CCR7 expression on gated lung tissue-resident CD4$^+$ T cells. Solid histograms show unstained cells in grey, PBS-treated WT in yellow, and CCL3$^{-/-}$ in peach, BeO-treated WT in brown, and CCL3$^{-/-}$ in purple. **(H)** Cumulative frequency of CCR7$^+$ CD4$^+$ T cells in the lungs of BeO-exposed mice. Error bars represent the mean ± SEM. 1-way ANOVA determined statistical significance. *P < 0.05, **P < 0.01, ***P < 0.001, ****P < 0.0001.

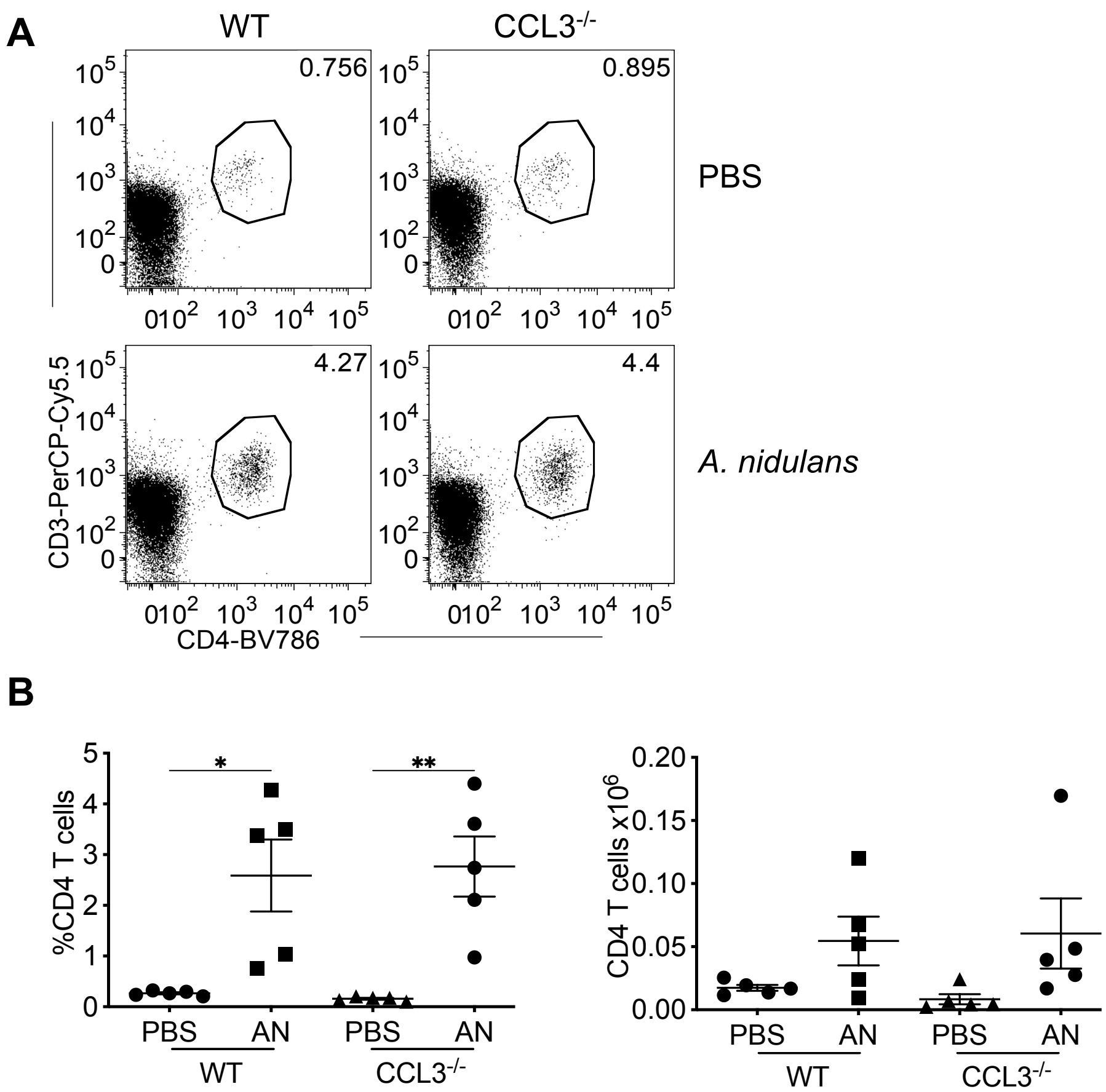

Supplementary Figure 4. Enhanced CD4$^+$ T cell responses in *A. nidulans* exposed HLA-DP2 Tg WT and CCL3$^{-/-}$ mice. Both WT and CCL3$^{-/-}$ mice were exposed to *A. nidulans* (1×10^8) spores/conidia on day 0, and the mice were sacrificed on day 14. A fluorescently labeled anti-CD45 mAb was injected intravenously 2 minutes before sacrifice to distinguish tissue-specific cells from circulating cells. On day 14, (**A**) flow cytometry plots show the frequency of CD4$^+$ T cells in the lungs of PBS or *A. nidulans*-exposed mice. (**B**) Graph plots show the frequency and number of CD4$^+$ T cells in the lungs of *A. nidulans*-exposed mice. Data are representative of the mean ± SEM of two independent experiments. 1-way ANOVA was used to determine statistical significance. *P < 0.05, **P < 0.01.

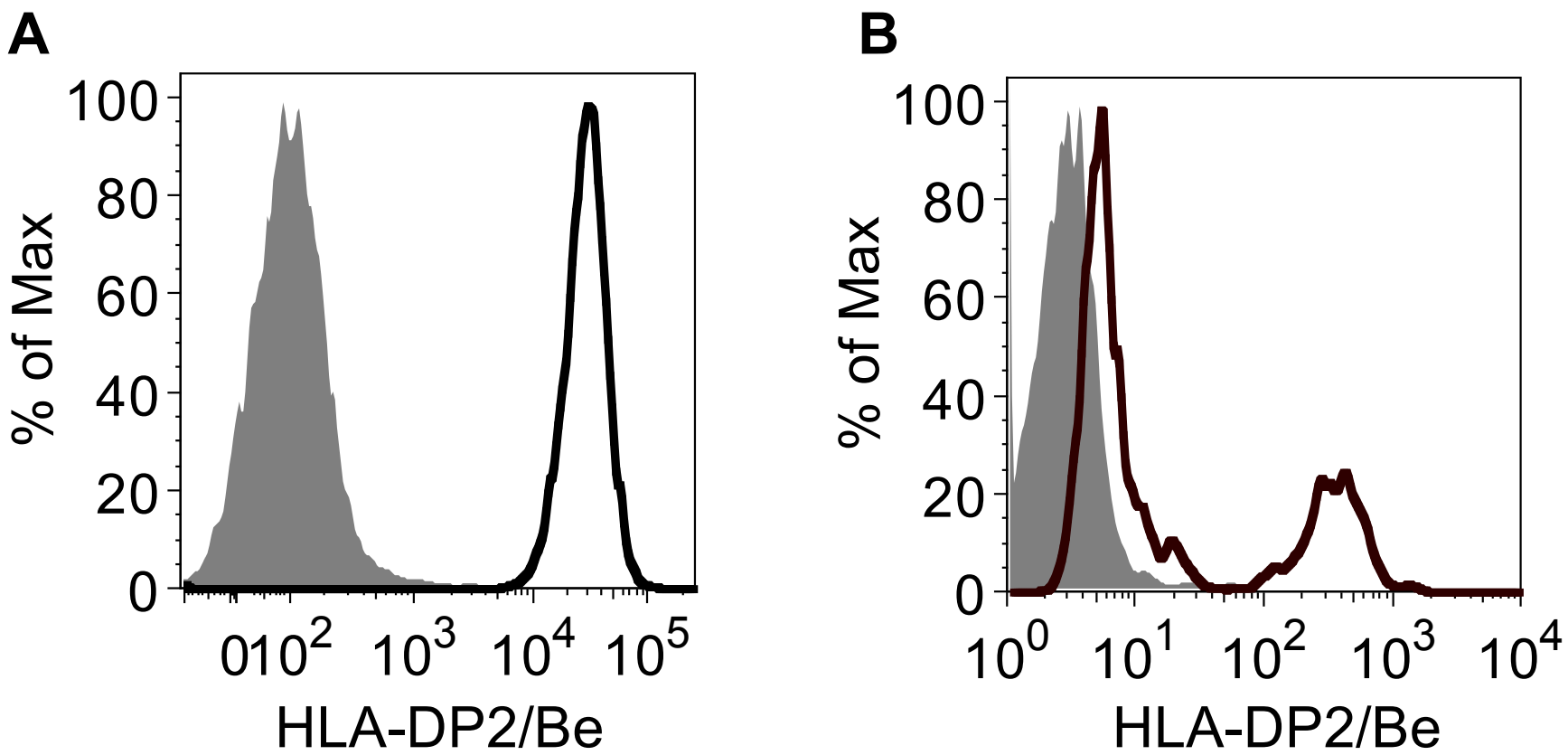

Supplementary Figure 5. Tetramer specificity validation. HLA-DP2-CCL4/Be and HLA-DP2-CLIP tetramer staining of **(A)** T cell hybridoma 8845-c3 that expresses a human TCR specific for the CCL4/Be neoantigen and restricted by HLA-DP2**,** and **(B)** gated $CD4^+$ T cells from the lungs of HLA-DP2 Tg mice, exposed to BeO under the standard regimen. Both tetramers were used at a final concentration of 20 μg/ml.

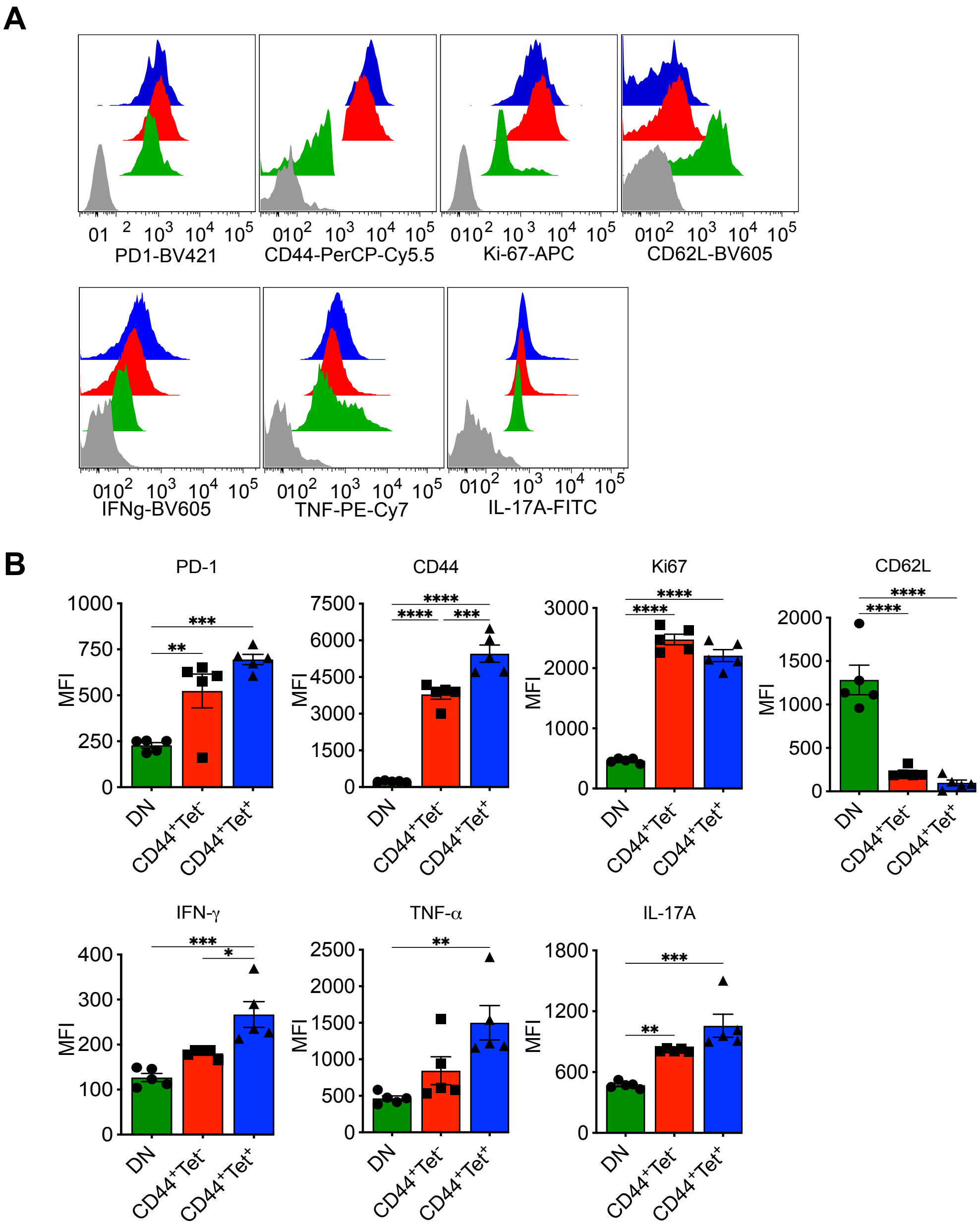

Supplementary Figure 6. Phenotypic and functional analysis of BeO-exposed lung tissue-resident CD4⁺ T cells. HLA-DP2 Tg FVB/N (WT) mice were intratracheally exposed to BeO (100 µg) on days 0, 1, 2, 14, 15, 18, and 19 and a single dose of LPS (10 µg) on day 14. On day 21, mice were injected intravenously with anti-CD45 mAb (5 µg/mouse) 2 minutes before sacrifice. Single cells were prepared from the lungs and stimulated with $BeSO_4$ (75 µM) for 4h in the presence of Brefeldin A (1X). The cells were stained with HLA-DP2-CCL4/Be tetramer (20 mg/ml) for 2h as described in the Materials and Methods. Subsequently, the cells were surface-stained, followed by intracellular staining to detect functional molecules. (**A**) Flow cytometric plots show cell surface expression of PD-1, CD44, and CD62L molecules and intracellular staining of proliferation marker, Ki-67, and the functional molecules, IFN-γ, TNF-α, and IL-17A. Histogram colors represent FMO control (grey), $CD44^-$ Tet^- $CD4^+$ T cells (DN, green), $CD44^+Tet^-$ $CD4^+$ T cells (Tet^-, Red), and $CD44^+Tet^+$ $CD4^+$ T cells (Tet^+, blue). (**B**) Bar graphs show the mean fluorescence intensity (MFI) of cell-surface and intracellular molecules for the various $CD4^+$ T cell subsets in the lungs of BeO-exposed mice. Data are representative of two independent experiments. 1-way ANOVA was used to determine statistical significance. *P < 0.05, **P < 0.01, ***P < 0.001, ****P < 0.0001.

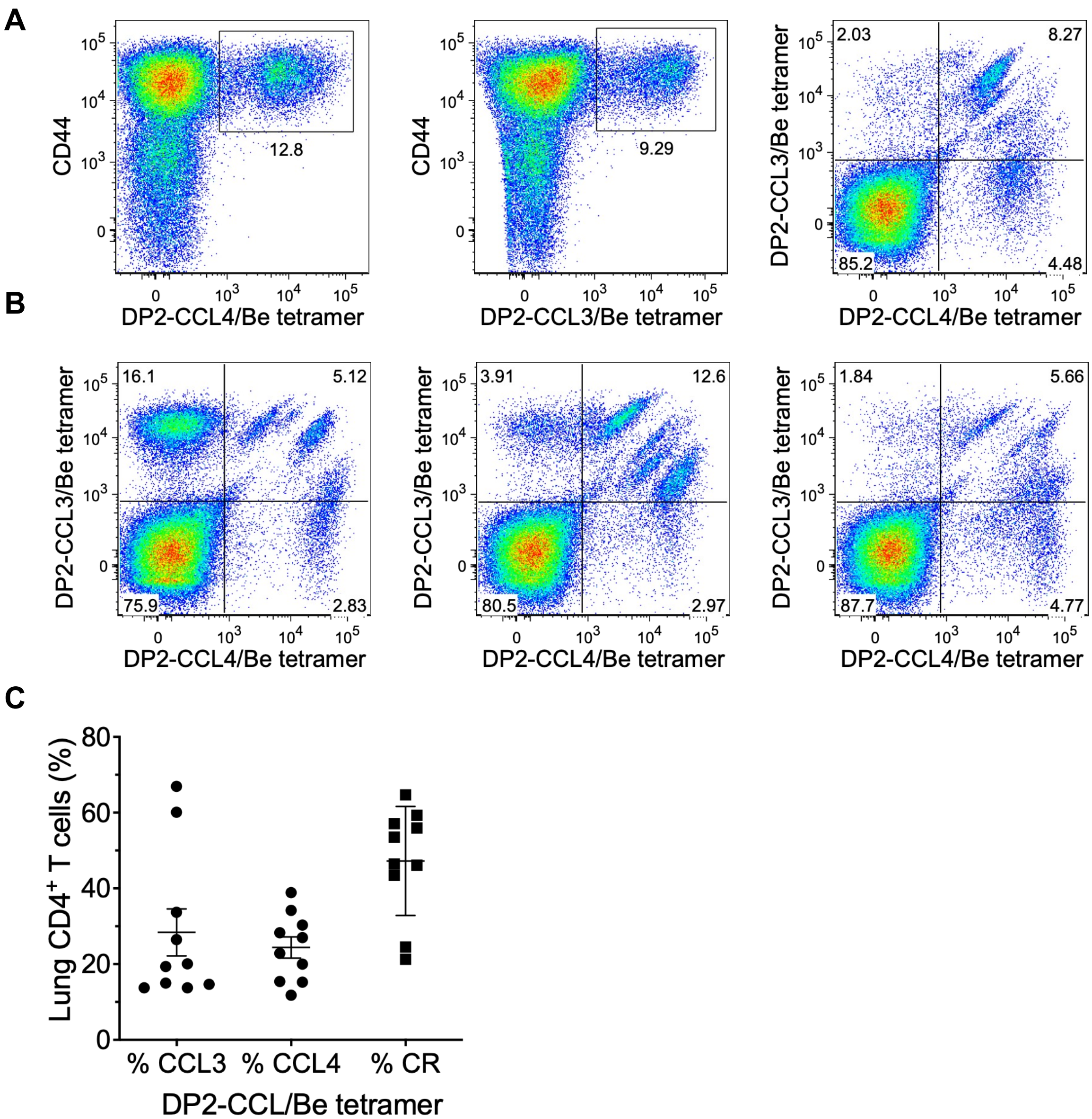

Supplementary Figure 7. BeO-exposure induced single or dual specificity antigen-specific (CCL3 and 4) CD4$^+$ T cells in the lungs. HLA-DP2 Tg mice (n=10) were sensitized and boosted with BeO (100 µg) on days 0, 1, 2, 14, 15, 18, and 19 and a single dose of LPS (10 µg) on day 14. On day 21, lung cells were stained with HLA-DP2-CCL3/Be and HLA-DP2-CCL4/Be tetramers labeled with different fluorophores. **(A)** Pseudocolor flow cytometry plots show representative DP2-CCL3/Be (left), and DP2-CCL4/Be (right) tetramer binding of CD4$^+$CD44$^+$ lung T cells. (**B**) Representative HLA-DP2 tetramer co-staining of lung CD4$^+$ T cells from BeO/LPS-exposed mice. (**C**) Summary of the overall percentage of lung CD4$^+$ T cells that bind individual tetramers, or are cross-reactive, i.e., bind both CCL/Be tetramers.

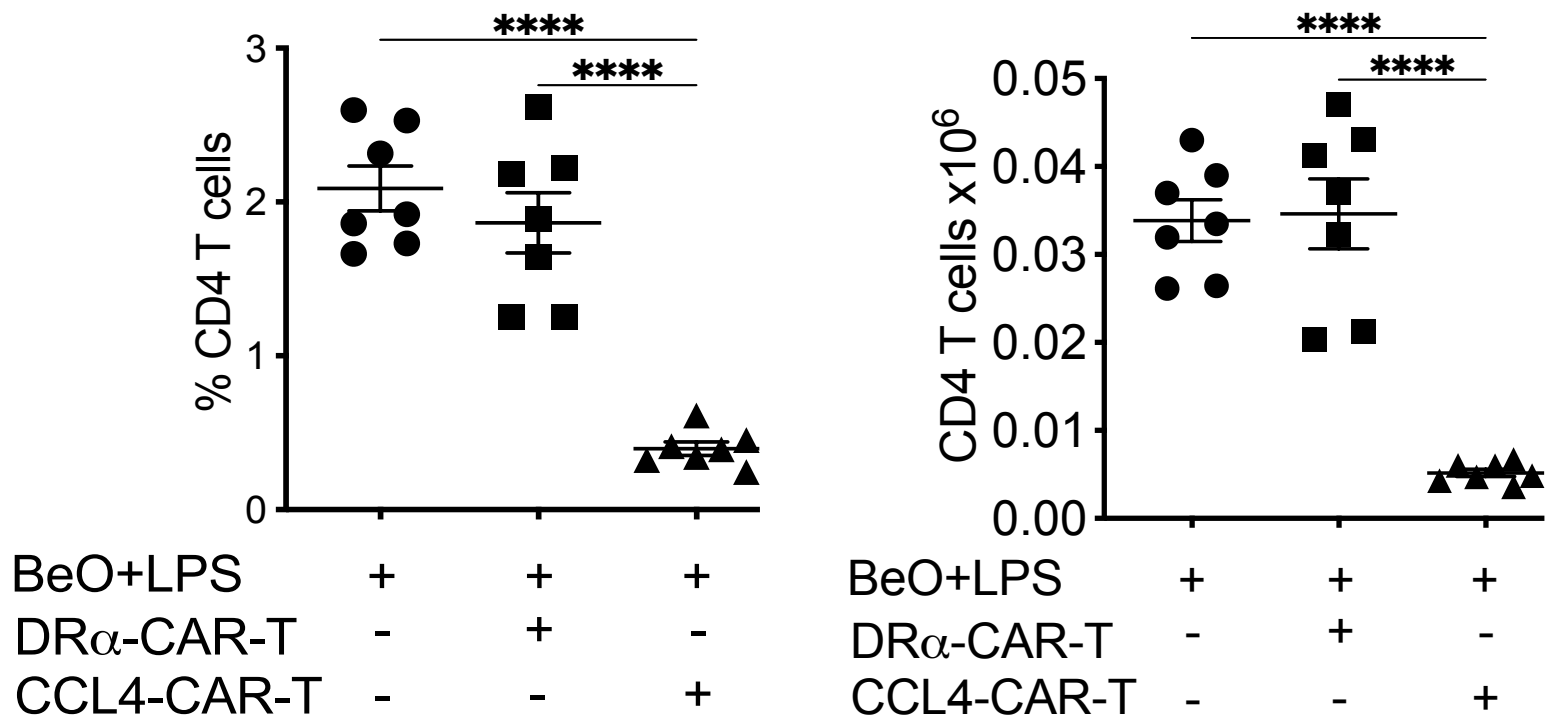

Supplementary Figure 8. Presence of $CD4^+$ T cells in the lungs of BeO/LPS-exposed mice treated with or without CAR-T cells. HLA-DP2 Tg mice were exposed to BeO on days 0, 1, 2, 14, 15, 18, and 19, and LPS (10 mg/ml) on day 14. Treated mice were given PBS or adoptively transferred with either control, DR-α-CAR-T cells, or CCL4/Be-CAR-T cells on day 8. Mice were sacrificed and examined on day 21. The frequency (left) and number (right) of $CD4^+$ T cells in the lungs of exposed mice are shown. Data are shown as mean ± SEM, pooled from two independent experiments. 1-way ANOVA was used to determine statistical significance. ***$P < 0.001$, ****$P < 0.0001$.
